# Kalata B1 Enhances Temozolomide Toxicity to Glioblastoma Cells

**DOI:** 10.3390/biomedicines12102216

**Published:** 2024-09-28

**Authors:** Samantha L. Gerlach, James S. Metcalf, Rachael A. Dunlop, Sandra Anne Banack, Cheenou Her, Viswanathan V. Krishnan, Ulf Göransson, Sunithi Gunasekera, Blazej Slazak, Paul Alan Cox

**Affiliations:** 1 Department of Biology, Dillard University, New Orleans, LA 70122, USA; 2 Brain Chemistry Labs, Institute for Ethnomedicine, Jackson, WY 83001, USA or jmetcal@bgsu.edu (J.S.M.); rachael@ethnomedicine.org (R.A.D.); sandra@ethnomedicine.org (S.A.B.); 3 Department of Biological Sciences, Bowling Green State University, Bowling Green, OH 43403, USA; 4 Department of Chemistry and Biochemistry, University of California, San Diego, CA 92093, USA; cher@ucsd.edu; 5 Department of Chemistry and Biochemistry, California State University, Fresno, CA 93740, USA; krish@csufresno.edu; 6 Department of Medical Pathology and Laboratory Medicine, University of California Davis School of Medicine, Davis, CA 95616, USA; 7 Pharmacognosy, Department of Pharmaceutical Biosciences, Uppsala University, Box 574, 751 23 Uppsala, Sweden; ulf.goransson@farmbio.uu.se (U.G.); sunithi.gunasekera@farmbio.uu.se (S.G.); blazej.slazak@farmbio.uu.se (B.S.); 8 W. Szafer Institute of Botany, Polish Academy of Sciences, 46 Lubicz, 31-512 Cracow, Poland

**Keywords:** cyclotides, glioblastoma, temozolomide, chemosensitize, chemotherapy, adjuvant therapy, *Viola*

## Abstract

Glioblastoma (GBM) is the most aggressive cancer originating in the brain, but unfortunately combination treatments with resection, radiation, and chemotherapy are relatively ineffective. Therefore, novel methods of adjuvant therapy are critically needed. Cyclotides are plant-derived circular peptides that chemosensitize drug-resistant breast cancer to doxorubicin. We analyzed naturally occurring and synthetic cyclotides (Cycloviolacin O3, Cycloviolacin O19, natural Kalata B1, synthetic Kalata B1, and Vitri E) alone and in co-exposure treatments with the drug temozolomide (TMZ) in human glioblastoma cells. The cyclotides were identified by UPLC-PDA and HPLC-UV. The synthetic Kalata B1 sequence was verified with orbitrap LC-MS, and structural confirmation was provided by NMR spectroscopy. The cyclotides displayed dose-dependent cytotoxicity (IC_50_ values 2.4–21.1 µM) both alone and as chemosensitizers of U-87 MG and T 98 cells to TMZ. In fact, a 16-fold lower concentration of TMZ (100 µM) was needed for significant cytotoxicity in U-87 MG cells co-exposed to synthetic Kalata B (0.5 µM). Similarly, a 15-fold lower concentration of TMZ (75 µM) was required for a significant reduction in cell viability in T 98 cells co-exposed to synthetic Kalata B1 (0.25 µM). Kalata B1 remained stable in human serum stability assays. The data support the assertion that cyclotides may chemosensitize glioblastoma cells to TMZ.

## 1. Introduction

The discovery of cyclotides dates back to 1973, when they were found to be the active peptides responsible for increasing uterine contractions and hastening labor within an ethnobotanical tea used by indigenous populations in Africa. Traditional healers of the Lulua Tribe in the Democratic Republic of the Congo boiled the aerial tissues of a plant they call Kalata-Kalata (*Oldenlandia affinis* (R & S) DC, Rubiaceae) and ingested the tea to facilitate labor [1]. One active compound elucidated from the plant extract is Kalata B1, the prototypic cyclotide. Cyclotides can be divided into three subfamilies, including bracelet, Möbius, and trypsin inhibitors, based on their structural and functional differences [2]. The circular variants can be found in five plant families and are common in violets (Violaceae), while they are sporadically found in genera of the Rubiaceae family. Within plants, cyclotides contribute to host defense and have been found to have insecticidal, antibacterial, and antifungal properties [3,4,5,6]. Cyclotides are resistant to thermal, chemical, and enzymatic degradation, which makes them promising candidates for therapeutic research [7,8].

One mechanism of cyclotide bioactivity includes the formation of monomer to tetramer pores on the phospholipid bilayer of cells. Cyclotides target the phosphatidylethanolamine (PE)-enriched head groups of phospholipids and disrupt lipid membranes via insertion [5,7]. The use of plants containing cyclotides in ethnomedical remedies has led to purified compounds which display both anti-cancer and anti-HIV activity [9,10]. We have found that cyclotides can chemosensitize HIV-infected cells, breast cancer cells, and glioblastoma cells to FDA-approved drugs [9,11,12].

Glioblastoma is a devastating and lethal brain cancer in children and adults, with a median survival rate of 9–16 months post diagnosis. The challenges associated with treatment include the lack of efficacy associated with resection, radiation, and chemotherapy [13,14]. The standard post-surgical drug regimen consists of temozolomide (TMZ); however, drug resistance is often rapid and only 50% of patients respond to TMZ. One significant challenge in the clinical treatment of patients suffering from glioblastoma is that high doses for prolonged periods (up to 49 days) may be required for efficacy, which can precipitously lead to TMZ resistance. After TMZ resistance develops, there are few, if any, other treatment options available since TMZ is considered the current “gold standard” of care [15,16].

Combination strategies with glioblastoma treatment have been hampered, at least in part, by the infiltrative nature of the tumor cells and the resistance and recurrence due to self-renewing tumor cell types such as glioblastoma stem cells, which escape chemo-radiotherapy and proliferate following treatment [15]. Our previous research indicates that a combination approach using cyclotides and FDA-approved drugs may chemosensitize breast cancer cells to doxorubicin [11], HIV-infected cells to saquinavir and T-20 [12], and glioblastoma cells to TMZ [9].

**Figure 1:**
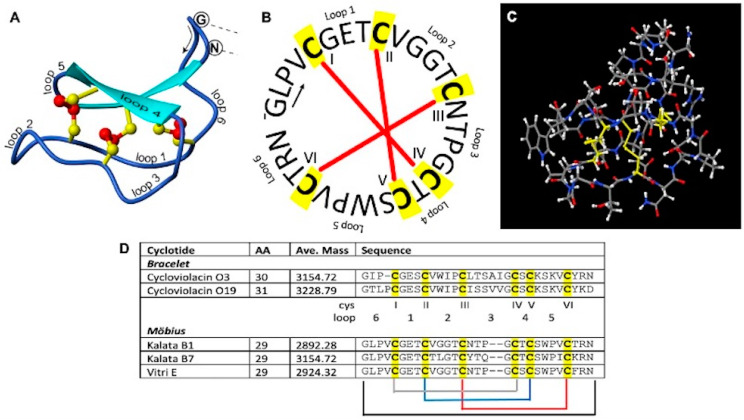
A representation of the structure and sequences of cyclotides. Panel (**A**) is a three-dimensional ribbon image of Kalata B1 (PDB 1NB1, SwissProt P56254), while panel (**B**) is a circular representation of Kalata B1. Panel (**C**) depicts a ball and stick model created in JSmol. The conserved cysteine residues are highlighted in yellow, while the disulfide bonds are depicted by the red bars in panel (**B**). Number of amino acids (AA), average molecular weights (ave. mass), and the sequence of amino acids are provided for cyclotides evaluated (panel (**D**)).

We evaluated five natural and one synthetic cyclotide in U-87 MG and T-98G human glioblastoma cells to evaluate their cytotoxicity alone and in combination with TMZ. Cycloviolacin O3 (CyO3) and Cycloviolacin O19 (CyO19) are bracelet cyclotides, whereas Kalata B1 (KB1), Kalata B7 (KB7), and Vitri E are Möbius cyclotides with 29–31 amino acids (Figure 1). Additionally, we identified the sequence of synthetic Kalata B1 using orbitrap LC-MS, verified its structure with NMR spectroscopy, and evaluated its stability in human serum. The current research significantly advances existing glioblastoma studies by demonstrating, for the first time to our knowledge, the dose-dependent cytotoxicity of several natural cyclotides (CyO3, CyO19, Kalata B7, and Vitri E) against U-87 MG human glioblastoma cells and the chemosensitizing property of the synthetically derived Kalata B1.

## 2. Materials and Methods

### 2.1. General Experimental Procedures

American Type Culture Collection (ATCC) provided the SH-SY5Y human neuro- blastoma (ATCC CRL-2266, PD21-26), U-87 MG human glioblastoma (ATCC HTB-14), and T-98G (ATCC CRL-1690) cells, as well as Eagle’s minimal essential medium (ATCC Cat. No. 30-2003). Dulbecco’s modified Eagle’s medium/Nutrient F-12 Ham (Cat. No. D8437), the Roche cell proliferation kit I (MTT) (Cat. No. 11465007001, Roche, Basel, Switzerland), dimethyl sulfoxide (DMSO) (Cat. No. D8418), NaCl (Cat. No. S7650), ammonium bicarbonate (NH4HCO3, Cat. No. A6141), acetonitrile (AcN, Cat. No. 34851), iodoacetamide (Cat. No. A3221), endoproteinase Glu-C (Cat. No. 1140399001), human serum isolated from male AB plasma (Cat. No. H4522), urea (Cat. No. U5128), and trichloroacetic acid (Cat. No. T6399) were purchased from Sigma-Aldrich (St. Louis, MO, USA). Dithiothreitol (Cat. No. 1610610) was purchased from Bio-Rad (Hercules, CA, USA). Temozolomide was provided by Selleck Chemicals, Houston, TX, USA (Cat. No. S1237). Trypsin (Cat. No. V5280) and chymotrypsin (Cat. No. V1061) were obtained from Promega (Madison, WI, USA). C18 (EC) SPE columns (Part No. 221-0100-B) were purchased from Biotage (Uppsala, Sweden). LiChroSolv water (Cat. No. 1.15333.1000), C18 Ziptips (Ref. No. ZTC185960), and the Ultrafree MCGV centrifugal 0.22 μm filters, Durapore RVDF (Ref. No. UFC30GVNB), were obtained through Millipore-Sigma, St. Louis, MO, USA. Nylon filters (0.2 μm, Cat. No. 7402-004) were supplied by Whatman GE Healthcare Life Sciences (Cleves, OH, USA). Ethanol (EtOH, Cat. No. 22032106) and trifluoroacetic acid (Cat. No. 04902-100) were purchased from Fisher Scientific (Waltham, MA, USA), while formic acid (Cat. No. 28905) and 11 mm, 600 μL plastic crimp/snap top autosampler vials (Cat. No. C4011-11) were obtained from Thermo Fisher Scientific. The Qubit standard protein assay kit (Cat. No. Q33212) was provided by Thermo Fisher Scientific. Each chemical was cell culture or reagent grade, and all digestive enzymes were sequencing grade.

### 2.2. Isolation and Purification of Plant Peptides

Cyclotides CyO3, CyO19, natural Kalata B1, and Vitri E were isolated using similar procedures described in a previous study [6]. CyO3, CyO19, and natural Kalata B1 were isolated from *V. odorata* L., while Vitri E was isolated from *V. tricolor* L. Plant material (leaves) of both species was bought from a commercial supplier, Alfred Galke GmbH (Gittelde, Germany). About 200 g of plant material was extracted with 2L of 60% methanol overnight. Subsequently, the extracts were diluted with water to below 20% methanol and loaded into a C18 low-pressure column (C18 spherical, 40–60 μm, 100 A, 40 g, Agela Technologies, Torrance, CA, USA) using a pump. The loaded columns were attached to a low-pressure chromatography system (Äkta FPLC, GE Healthcare, Chicago, IL, USA) operated with a flow rate of 20 mL min^−1^ and a gradient of 10–95% ACN, 0.1% TFA, and one min fractions were collected. The fractions containing the cyclotides were identified using LCQ electrospray ion trap MS (Thermo Finnigan, San Jose, CA, USA), operated in the positive-ion mode with direct injection. The peptides were purified from their respective fractions using HPLC. Kalata B7 was isolated from *Geophilia repens* (Rubiaceae) during a previous study [17].

CS Bio Co. (Menlo Park, CA, USA, 94025) synthesized 30 mg of Kalata B1 (Sample No. CS9566, Lot No. X477) with a purity of 97.51%, as analyzed by Agilent (Santa Clara, CA, USA) 1290 HPLC using a Phenomenex Luna C 18 5 µm 100 Å, 4.6 × 250 mm column (25 °C). The HPLC method involved 30–60% Buffer B for 20 min (Buffer A = 0.1% TFA in H_2_O and Buffer B = 0.1% TFA in ACN) with a flow rate of 1 mL/min., monitored at a wavelength of 214 nm. A linear peptide was synthesized, cyclized, and properly folded using head-to-tail lactam bonding and natural oxidation (Figure S1) and the identity was confirmed via comparison with the natural Kalata B1.

### 2.3. Verification of Synthetic Kalata B1

#### 2.3.1. UPLC-MS Synthetic Kalata B1 Verification

Purified CyO3, CyO19, natural Kalata B1, Kalata B7, and Vitri E were subjected to ultraperformance liquid chromatography coupled with photodiode array detection (UPLC-PDA) and reverse-phase high-performance liquid chromatography with UV detection (HPLC-UV). During UPCL-PDA, 5 µL injections (10% (*v*/*v*) ethanol (EtOH) in water) were loaded onto a C 18 LC Column (Kinetex Core-Shell) of 100 mm × 2.1 mm, 1.7 µM, 100 Å. The flow rate was maintained at 0.65 mL/min using a Waters Acquity Ultraperformance LC (Milford, MA, USA). The solvents were as follows: water + 0.1% (*v*/*v*) trifluoroacetic acid (A) and acetonitrile + 0.1% (*v*/*v*) trifluoroacetic acid. The gradient was set from 0–10% B for 1 min, then 10–60% B for 6 min, followed by 60–85% B for 2 min. The column temperature was set at 40 °C, while the sample temperature was 7 °C. During the UPLC-PDA analysis, the same gradient and temperatures were used; however, the loop was switched from partial to a full loop. The following *m*/*z* ratios were monitored: TMZ (195.06), synthetic and natural Kalata B1 (964.77), CyO3 (1051.80), CyO19 (1076.94), and Vitri E (975.45). Chromatograms were evaluated using Empower 3 imaging software, version 7.50.00.00.

#### 2.3.2. Orbitrap Exploris 480 Liquid Chromatography-Mass Spectrometry

Synthetic Kalata B1 peptide was modified and digested with endoGluC and trypsin before being cleaned up with C18 zip tips, according to Gerlach and colleagues [9]. The dried residue was resuspended with 20 μl Direct Q water + 0.1% (*v*/*v*) formic acid for mass spec analysis. The samples were analyzed using an Orbitrap Exploris 480 mass spectrometer (Thermo Scientific) and an Easy-nLC 1200 (Thermo Scientific) nano liquid chromatography system. For the LC separation, samples (1 μL) were injected onto a nanoEase C18 column (Waters MZ HSS T3 column, 100 Å, 1.8 μm, 75 μm × 150 mm) at a flow rate of 20 μL per minute in a volume of 6μL and maximum pressure of 980 bar with solvents of water (A, LiChrosolv, EMD Millipore) + 0.1% formic acid (Ricca Chemicals, Arlington, TX, USA) and 80% acetonitrile in water (LiChrosolv, EMD Millipore) + 0.1% formic acid. The samples were separated for mass spectrometric analysis using a gradient of 5–100% B over 12 min, maintaining 100% B for a further 8 min with a flow of 300 nL/min and a total run time of 20 min. The analytical column equilibration was 10 μL, with a maximum pressure of 500 bar. The flush volume was set at 100 μL. Samples were analyzed by tSIM-MS/MS, modifying several parameters to improve detection of the peptides. The source was maintained at a spray voltage of 1.4 kV and capillary temperature of 250 °C. After digestion, for the optimal detection of the two Kalata B1 peptides, the following methods were used.

*Peptide 1 (NGLPVCGE)*: A tSIM method was set up with a default charge state of 2, but it also included a charge state of 1, an isolation window of *m*/*z* = 2, an orbitrap resolution of 480,000, 1 microscan, an RF lens setting of 50%, and 20 dependent scans. tSIMs of 845.3821 (z = 1) and 423.1947 (z = 2) were used to isolate peptides before MS/MS. These scans were performed with an isolation window of *m*/*z* = 2, a stepped energy mode of 15, 30, 45%, an orbitrap resolution of 480,000, 1 microscan, and an expected LC peak width of 15. All other parameters were set at the manufacturers’ default.

*Peptide 2 (RTCVPWSCTCGPTNCTGGVCT)*: Mass spectrometric analysis was performed the same as for peptide 1 with the following changes. During the tSIM, charge states of 2 and 3 were assessed with an RF lens of 125% and tSIMs of 1215.9894 (z = 2) and 810.9954 (z = 3). The MS/MS parameters were the same as for peptide 1, with stepped energies of 20, 40, 60%. After analysis, all peptides were identified and sequenced by hand in Freestyle software version 1.7 (Thermo Scientific).

#### 2.3.3. NMR Spectroscopy

Approximately 10 mg of the peptide was prepared in 600 µL in 90% H_2_O/10% D_2_O. All the NMR experiments were performed at a pH of 3.6 and a sample temperature of 303 K. The NMR experiments were performed on a JEOL 600 MHz spectrometer (Fresno State University, Fresno, CA, USA) equipped with the SuperCOOL cryogenic (liquid N_2_) probe with a triple-axis pulse-field gradient. The following two-dimensional (2D) NMR experiments were collected: ^1^H-^1^H double quantum filtered correlation spectroscopy (DQFC) [18], ^1^H-^1^H total correlation spectroscopy (TOCSY, 80 ms with a DIPSI spin-lock) [19], ^1^H-^1^H nuclear Overhauser effect spectroscopy (NOESY, 150 ms), and ^1^H-^1^H rotating frame nuclear Overhauser effect spectroscopy (ROESY, 200 ms with adiabatic spin-lock with a 6.0 kHz) [20,21,22].

Typically, the time domain data were collected with 4096 × 256 (t_2_ × t_1_) complex points in a phase-sensitive mode, and 128 transients were collected per t_1_ increment with a relaxation delay of 2.0 s. The chemical shifts were referenced using sodium trimethyl-silyl propane sulfonate (DSS) (TMS) as the internal standard. Three-bond ^1^H-^1^H coupling constants were measured from one-dimensional spectra. NMR data were processed using a combination of JEOL Delta (zero-filled twice, sine-squared window functions, and complex Fourier transform) and saved in the Sparky [23,24] format. The integration function in Sparky was performed to obtain the volume for each NOE cross peak. Finally, the chemical shifts and NOE cross-peak volume were exported from Sparky for structure calculation.

#### 2.3.4. The Ensemble of NMR Structures

The structure calculation of Kalata B1 was performed using the program “combined assignment and dynamics algorithm for NMR applications” (CYANA) [25]. The ensemble of representative structures was generated using a combination of NOESY cross-peaks, chemical shifts, and three-bond (^3^J_HNα_) coupling constants. In general, the structure calculation started with 1000 randomly generated structures. Two thousand and forty-eight randomly generated structures underwent simulated annealing using 16,384 molecular dynamics steps within the provided parameters. Two individual structure calculations were performed with additional parameters added on the next calculation as follows: (1) using torsion angle restraints generated based on the amino acid sequence and cis proline omega torsion angle for the P24 and (2) adding upper limit distance constraints between the protons generated from the NOE cross peak volume. When generating the upper limit distance constraints, an average upper distance limit of 4.2 Å was used. The ‘*distance modify*’ command in CYANA was used to remove redundant and unnecessary distance constraints and apply the necessary pseudo-atom corrections. One hundred forty-nine upper limit distance constraints were generated and used for the structure calculation. The structure calculation selected the 20 lowest energy structures as the structural representation within the given parameters. The structure analysis was performed using PyMOL (the PyMOL Molecular Graphics System, Version 2.5.4, Schrodinger, LLC, New York, NY, USA).

### 2.4. Quantifying Cyclotides for Evaluation in Bioactivity Assays

Freeze-dried samples of CyO3, CyO19, Kalata B1, Kalata B7, and Vitri E (~0.5–2.0 mg) were dissolved into 10% DMSO and quantified following the protocols described by Gerlach and colleagues [9]. The concentrations of the peptides were determined spectrophotometrically using extinction coefficients determined by the ExPASy Protparam tool (https://web.expasy.org/protparam/, accessed on 14 August 2024) as follows: CyO3 (7365 mol^−1^cm^−1^), CyO19 (7365 mol^−1^cm^−1^), synthetic and natural Kalata B1 (5875 mol^−1^cm^−1^), Kalata B7 (7365 mol^−1^cm^−1^), and Vitri E (5875 mol^−1^cm^−1^). In addition to quantification via UV absorbance at 280 nm in a Nanodrop 2000 spectrophotometer (ThermoScientific), the peptides were also quantified as previously described by Gerlach et al. 2022 [9], using Qubit 3.0 fluorometer (Thermo Fisher Scientific Invitrogen Life Technologies) protein quantification assays (Cat. No. Q33212).

### 2.5. Cell Culture

SH-SY5Y human neuroblastoma cells (population doubling (PD) 20−24), T-98G, and U-87 MG human glioblastoma cells were removed from cryogenic storage, thawed rapidly in a water bath (37 °C) and reconstituted in 75 cm^2^ flasks. For SH-SY5Y, the following media were used: Dulbecco’s modified Eagle’s medium/Nutrient F12 Ham (Sigma-Aldrich, Cat. No. D8437) containing 10% heat-inactivated fetal bovine serum, 4 mM L-glutamine, 100 U/mL penicillin, and 100 μg/mL streptomycin. For U-87 MG and T-98G Eagle’s minimal essential medium (ATCC: The Global Bioresource Center, Cat. No. ATCC 30-2003), containing 10% heat-inactivated fetal bovine serum, 4 mM L-glutamine, 100 U/mL penicillin, and 100 μg/mL streptomycin were used.

Cells were incubated at 37 °C in a humidified atmosphere of 5% CO2 and 95% air until recovered and ready for passaging (approximately 4 days). At between 80% and 90% confluence, the cells were trypsinized with warm (37 °C) sterile 0.5% Trypsin-EDTA (Thermo Fisher, Cat. No. 15400054) and passaged into 175 cm^2^ flasks, where they were left to multiply until they reached 80−90% confluence. To plate cells: cultures were trypsinized, resuspended in 20 mL sterile PBS, counted using a cell counter (Olympus cell counter model R1), and plated in 96-well plates at an optimal density of 6000 cells/well in the designated culture media. The cells were left to adhere overnight before treatment the following day, when the culture medium was removed and replaced with the treatment medium. All incubations were conducted in triplicate wells and repeated at least three times (n = 3).

### 2.6. Phase Contrast Microscopy

U-87 MG cells were plated into 96-well plates at 6000 cells/well, allowed to adhere overnight, then the medium changed to the following: (A) no treatment/control; (B) 150 µM temozolomide (TMZ); (C) synthetic Kalata B1 0.5 µM; and (D) 150 µM TMZ + 0.5 µM synthetic Kalata B1. The cultures were incubated for 72 h. The cultures were imaged on an Olympus IX83 inverted microscope using phase-contrast with an Olympus XM10 camera at 20× magnification (Tokyo, Japan). Images were acquired in cellSens Standard, then imported into Adobe Photoshop 23.4.2, where they were adjusted for exposure using auto “Levels”, sharpened using Topaz Laboratories Sharpen AI version 2.1.7 and cropped for consistency and to enable ease of comparison.

### 2.7. Determination of Half-Maximal Inhibitory Concentration (IC_50_ Values) in TMZ and Cyclotides

To calculate TMZ IC_50_ values in each cell line, TMZ was dissolved in DMSO at a stock concentration of 103 mM, which was then diluted into culture medium on the day of use, with the DMSO final concentration always less than 0.1%. The following concentrations of TMZ were used in the experiments: 0.5, 5, 50, 500, 2500, 5000 μM. The media were sterilized for each treatment media with a 0.22 μm filter before they were added to cell cultures. To determine the IC_50_ values for each cyclotide in each cell type, cyclotides were dissolved into 10% DMSO and then dilutions used to screen cyclotides with a final DMSO concentration of less than 0.1% The following concentrations of cyclotides were used: 0.1, 1, 5, 10 μM. For co-exposure experiments, a simultaneous exposure of cyclotide and TMZ at each respective dose was added to cell cultures (100 μL final volume per well).

### 2.8. MTT [3-(4,5-Dimethylthiazol-2-yl)-2,5-diphenyltetrazolium bromide] Assay

In accordance with the methods developed by Mosmann and colleagues [26], the MTT assay was conducted using the Roche cell proliferation kit (Cat. No. 11465007001). Briefly, cells that are active can cleave MTT. The MTT is originally a yellow tetrazolium salt that undergoes a colorimetric change to purple formazan crystals, and the change in absorbance can be measured as an indicator of cell viability/proliferation. For these experiments, cells were plated into 96-well flat-bottom plates, as described in the cell culture above. After 24 h, the medium was replaced with 100 µL treatment medium consisting of either the control, TMZ, cyclotide alone, or a co-exposure treatment of TMZ + cyclotide. To align with our previous work [9], experiments were conducted with a 72 h time frame for cytotoxicity evaluation.

The concentrations of cyclotides alone were as follows: 0, 0.1, 1, 5, and 10 µM of either CyO3, CyO19, Kalata B1, Kalata B7, and Vitri E. The maximum dose of 10 µM was used because previous research [9] indicated this concentration provided sufficient cell death while preserving limited quantities of purified cyclotides. After a 72 h incubation period, plates were removed from the incubator and 10 µL of MTT was added to each well. The plates were returned to the incubator for an additional 4 h, after which 100 µL of a solubilization solution was added to each well. The plates were returned to incubation overnight, and the next day absorbances were read at 570 nm on a Molecular Devices FlexStation 3 using SoftMax Pro Microplate Data Acquisition and Analysis Software (version 7.0.2). All data were expressed as a percent of controls in a normalized response, and all incubations were conducted in triplicate wells (technical replicates) and in triplicate, independent incubations (n = 3).

### 2.9. IC_50_ Value Calculations and Statistical Analysis

We calculated the IC_50_ values with a best fit method using nonlinear regression analysis with dose-dependent inhibition and an inhibitor versus normalized response. MTT assay results were analyzed statistically using one-way analysis of variance (ANOVA) and Tukey’s post hoc analysis, where *p* < 0.05 was considered significant. All data were evaluated using GraphPad Prism for Mac OS (version 9.5.1). For each calculation, at least three independent experiments with 3 biological replicates for each experiment were performed.

### 2.10. Human Serum Stability Assay

Established protocols [27,28] were used to analyze peptide stability in human serum isolated from male AB plasma (Sigma-Aldrich). Briefly, thawed plasma was centrifuged for 10 min at 15,000× *g* to concentrate lipids. The supernatant was carefully removed with a Pasteur pipette and incubated for 15 min at 37 °C, 5% CO_2_. Stock synthetic Kalata B1 and TMZ (300 µM) were diluted 10 times with warmed plasma or warmed PBS for 0, 3, 8, and 24 h. Controls were synthetic Kalata B1 or TMZ in PBS that were incubated in parallel with human serum test compounds. Serum proteins were denatured with urea (final concentration of 3M) at 4 °C for 10 min., at each time point. Afterwards, trichloroacetic acid (7% final *v*/*v*) was added for 10 min to precipitate the proteins. Each sample was centrifuged for 10 min at 15,000× *g*; the supernatant was removed an evaluated in triplicate using UPLC-MS.

## 3. Results

### 3.1. Verification of Synthetic Kalata B1

#### 3.1.1. UPLC-MS Synthetic Kalata B1 Verification

**Figure 2:**
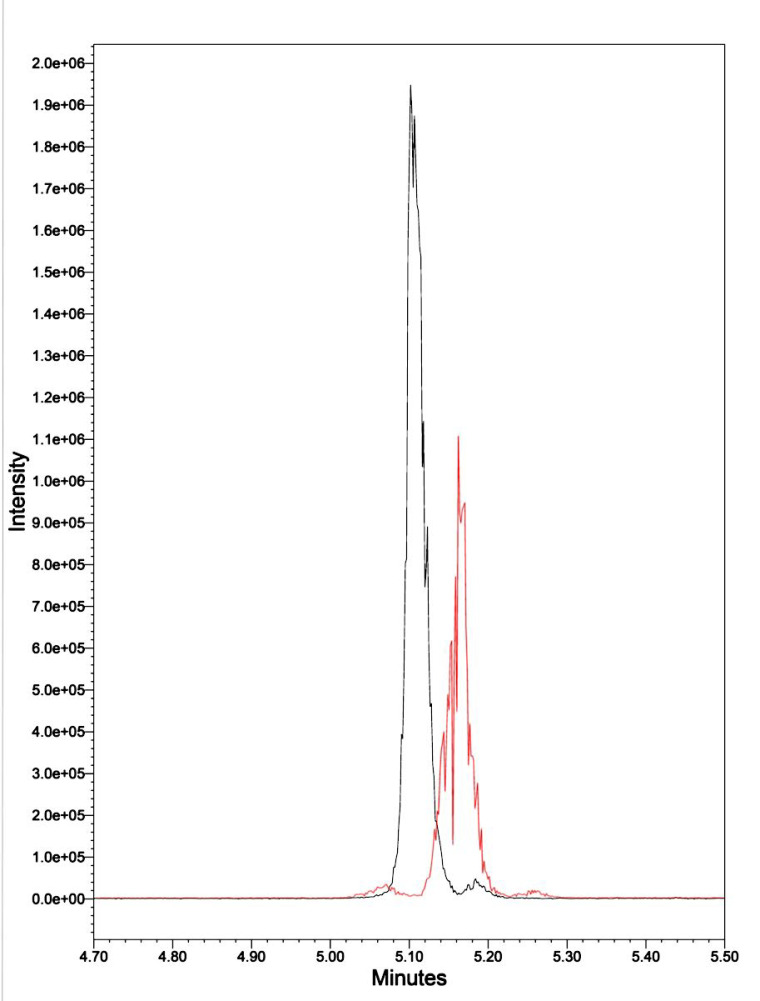
UPLC-MS of synthetic Kalata B1 (black peak) and natural Kalata B1 (red peak), each with a *m*/*z* of 964.77 and an elution time of 5.05–5.25 min.

In previous research [9], we demonstrated that our stock of purified natural Kalata B1 was 95.4% pure. We commissioned CSBio (Menlo Park, CA, USA) to synthesize 30 mg of synthetic Kalata B1, which is documented at 97.51% pure. They synthesized a linear cyclotide, cyclized it, and folded it using head-to-tail lactam bonding and natural oxidation (Figure S1). The present UPLC-MS analyses of both synthetic and natural Kalata B1 indicated they had an elution time of 5.05–5.25 min., and both have a *m*/*z* of 964.77. There was a slight shift in the elution time, which was attributed to matrix interference (Figure 2).

#### 3.1.2. Orbitrap Analysis of Exploris 480 Mass Spectrometry–Liquid Chromatography

**Figure 3:**
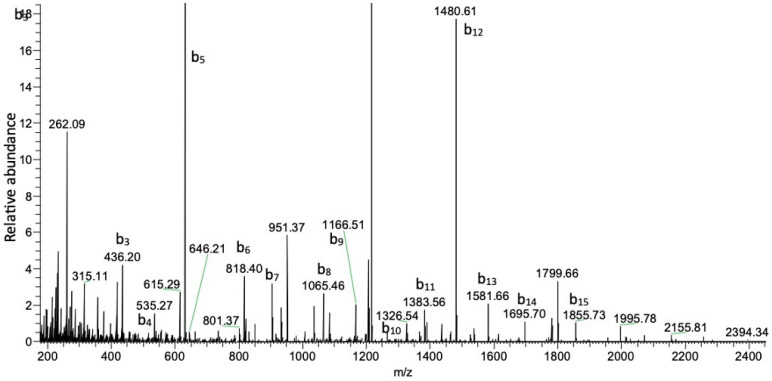
MS/MS analysis of peptide 2 (RTCVP) with a mass of *m*/*z* 1215.9891.

The Orbitrap analysis of the alkylated and enzymatically digested peptides revealed the presence of ions consistent with digested Kalata B1 (e.g., Figure 3). The assessment of the masses observed showed good consistency with the exact masses expected, with *m*/*z* values of 845.3799 and 1215.9891 observed for peptides 1 and 2, respectively. Sequence analysis of the two peptides showed daughter ions consistent with the expected sequences (Table 1 and Table 2). For peptide 1, all the amino acids could be found in this 8-mer peptide (Table 1), whereas only 19 of the 21 amino acids could be sequenced for peptide 2 (Table 2). However, given the expected vs. observed *m*/*z* ratios of the parent peptides (845.3821 vs. 845.3799, peptide 1 and 1215.9894 vs. 1215.9891, peptide 2), both peptides match Kalata B1, with 93% coverage of the peptide according to Proteome Discoverer (Thermo Scientific) assessment of the sequence.

#### 3.1.3. The Structural Characteristics of Kalata B1 Using NMR Spectroscopy

**Figure 4:**
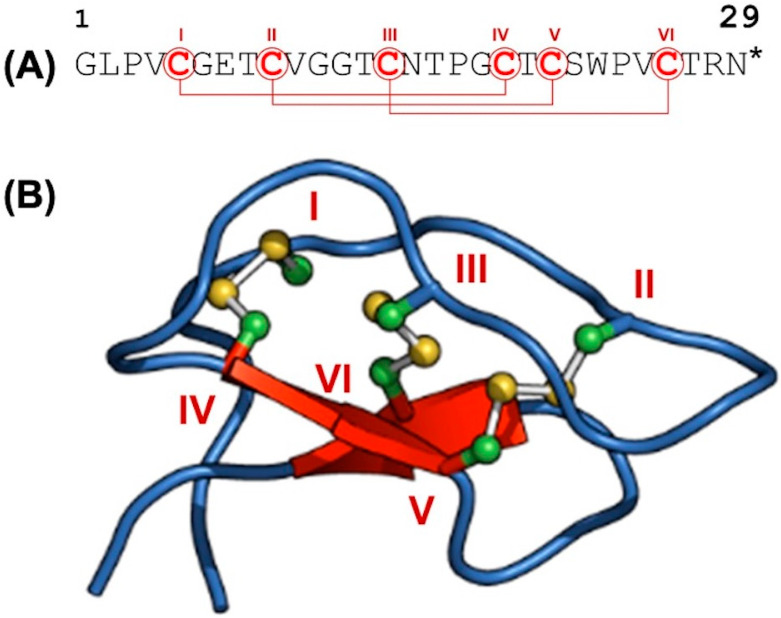
The structural features of Kalata B1. (**A**) The amino acid sequence of Kalata B1. (**B**) An ensemble of averaged structure of Kalata B1 determined using NMR spectroscopy. The positions of the cysteines (labeled I through IV) and the corresponding disulfide linkages are shown. The * indicates a point of known cyclization between the glycine (C) and Asparagine (N) residues.

The DQFC, TOCSY, and NOESY spectra were used for the sequence-specific backbone chemical shift assignment of Kalata B1 (Figure 4A). A representative three-dimensional structure determined by NMR spectroscopy is also shown (Figure 4B). The six cysteine residues (I to VI) and the corresponding disulfide bonds in the three-dimensional fold of the synthetic Kalata B1 are indicated. All the proton chemical shifts of Kalata B1 were assigned (Table 3), except for the protons on the end of the guanidino group from Arg 28, the thiol protons from the six cysteine residues, and the protons from the carboxamide group of Asn 15 and Asn 29. Table 1 also lists the three-bond ^1^H-^1^H coupling constant (^3^J_NH-CαH_).

After assigning the backbone chemical shift and confirming the cyclic peptide nature of Kalata B1 via the NOE cross peak between H^α^ of Asn 29 to the H^N^ of Gly 1 (Figure S2b), the DQFC and TOCSY were used to complete the chemical shift assignment of the side chains. Kalata B1 has three proline residues at positions 3, 17, and 24. Proline can exist as a cis or trans isomer due to the peptide bond linkage, which can be identified via NMR [29]. Pro 3 and Pro 17 were present as trans isomers, as indicated by the NOE cross peak between the H^δ^ of the proline to the H^α^ of the preceding residue on its N-terminus side (Figure S2c). On the other hand, pro 24 was present as a cis isomer, as evidenced by the NOE cross peak between the Hα of the proline and the H^α^ of the preceding residue on its N-terminus side (see Figure S2c). Initial structures were generated without the NOE distance constraints to convey the cyclic nature of Kalata B1 via the NMR data. An evolution of the structure generation process is illustrated (Figure S3), showing the structure before and after adding the NOE distance constraints.

The 20 Kalata B1 structures generated by CYANA using NOE distance constraints have no distance violation greater than 0.3 Å (Figure S4a). The average backbone RMSD to mean was 0.56 ± 0.25 Å (0.27…1.53 Å; 20 structures), and the average heavy atom RMSD to mean was: 0.87 ± 0.29 Å (0.59…2.02 Å; 20 structures). A three-dimensional alignment of the structure of Kalata B1 presented here has an RMSD of 1.18 Å, with reference to the solution structure of Kalata B1 from the natural resources (PDB 2khb).

### 3.2. Determination of IC_50_ Values in TMZ and Cyclotides

The IC_50_ values of natural and synthetic cyclotides, as well as TMZ, are provided in Table 4. As illustrated, each cyclotide (CyO3, CyO19, natural Kalata B1, synthetic Kalata B1, and Vitri E) showed much lower IC_50_ values compared to the TMZ IC_50_ values. Synthetic Kalata B1 was approximately 2-fold more potent in the T-98G cells compared to the U-87 MG cells. Natural Kalata B1 (IC_50_ = 7.9 µM) was comparable to the synthetic Kalata B1 (IC_50_ value = 5.4 µM) in U-87 MG cells. The IC_50_ values were calculated using nonlinear regression analysis with dose-dependent inhibition and an inhibitor vs. normalized response, as calculated by GraphPad Prism 9.5.1. For details of cell culture, see Materials and Methods. ^a^ represents data previously published [9].

**Figure 5:**
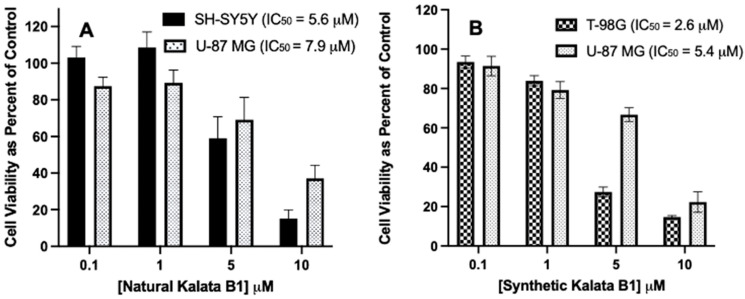
Effect of natural and synthetic Kalata B1 on cell viability in SH-SY5Y, U-87MG, and T-98G cells cultures. The IC_50_ values for the cyclotides were determined using MTT assay after incubation with cells at increasing concentrations (0.1, 1, 5, and 10 μM) as compared to the control. Panel (**A**): SH-SY5Y and U-87 MG cells, and panel (**B**) T-98G and U-87 MG cells. Cultures (6000 cells per well in a 96-well plate), were treated with the cyclotide indicated, for 72 h, then MTT measured. Cell viability was calculated as a percentage of control cultures. IC_50_ values were calculated using the Best Fit method (See Table 1). Incubations were conducted in 96-well plates in triplicate wells (technical replicates) and as three independent incubations (biological replicates, n = 3). Data are expressed as mean ± standard error of the mean (SEM). The results are graphed in GraphPad Prism version 9 for iOS.

The IC_50_ values of TMZ, natural cyclotides (CyO3, CyO19, Kalata B1, Kalata B7, and Vitri E), and the synthetic cyclotide Kalata B1 were evaluated in T-98G human glioblastoma, U-87 MG human glioblastoma, and SH-SY5Y human neuroblastoma cell cultures using MTT assays by plating cells in 96-wells with various concentrations of cyclotides and a 72 h incubation period. The IC_50_ value can be defined as the concentration of the drug (cyclotide), whereby 50% of the cells survive, and cell viability was then expressed as a percentage of control (cells incubated with DMSO < 0.01%). As illustrated in Figure 5, lower concentrations of TMZ were required to obtain an IC_50_ value in T-98G cells (1160.0 µM) compared to U-87 MG cells (1617.0 µM).

**Figure 6:**
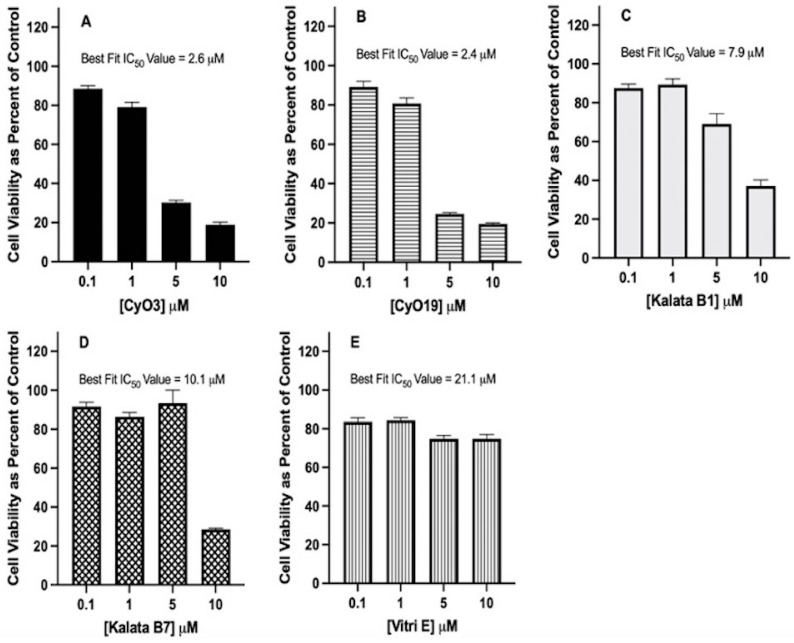
Effect of increasing the concentration of naturally occurring cyclotides on cell viability in U-87 MG cells. IC_50_ values for cyclotides were determined using the MTT assay after incubating U-87 MG cell cultures with increasing concentrations (0.1, 1.0, 5.0, and 10 μM) or with a control of DMSO only. Panel (**A**): CyO3, panel (**B**): CyO19, panel (**C**): Kalata B1, panel (**D**): Kalata B7, and panel (**E**), Vitri E. U-87 cells (6000 cells per well in a 96-well plate) were treated with cyclotides, as indicated, for 72 h, then MTT measured. Cell viability was calculated as a percentage of control cultures. IC_50_ values were calculated using the Best Fit method (See Table 1). Incubations were conducted in 96-well plates in triplicate wells (technical replicates) and as three independent incubations (biological replicates, n = 3). Data are expressed as mean ± standard error of the mean (SEM). Results are graphed in GraphPad Prism version 9 for iOS.

Increasing concentrations of plant-derived cyclotides (CyO3, CyO19, Kalata B1, Kalata B7, and Vitri E) were evaluated in MTT assays to determine the IC_50_ values in U-87 MG glioblastoma cell cultures. The cyclotides exhibited dose-dependent cytotoxicity, and the Best Fit method for IC_50_ value was calculated with nonlinear regression analysis using dose-dependent inhibition with an inhibitor vs. normalized response and confidence intervals of 95%, as calculated by GraphPad Prism 9.0 for iOS. Cyclotides exhibited a dose-dependent cytotoxicity, with IC_50_ values ranging from 2.4 to 21.1 µM. The cyclotides demonstrating the most to least potent cytotoxicity were as follows: CyO19 (IC_50_ value = 2.4 µM), CyO3 (IC_50_ value = 2.6 µM), Kalata B1 (IC_50_ value = 7.9 µM), Kalata B7 (IC_50_ value = 10.1 µM), and Vitri E (IC_50_ value = 21.1 µM) (Figure 6).

**Figure 7:**
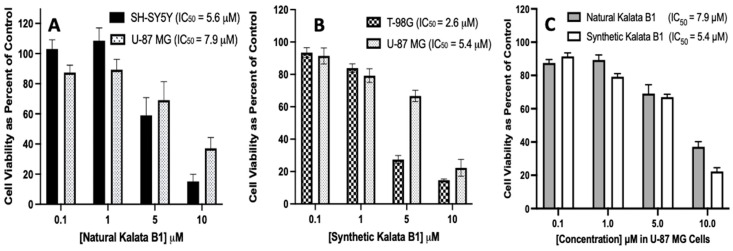
Comparison of IC_50_ values for natural and synthetic Kalata B1, as determined in SH-SY5Y, U-87 MG, and T-98G human cell cultures. Panel (**A**) includes SH-SY5Y and U-87 MG cells, panel (**B**) includes T-98G and U-87 MG cells, and panel (**C**) is natural Kalata B1 and synthetic Kalata B1 with concentrations of 0.1, 1, 5, and 10 μM as compared to the control. Cultures were plated in 96-well pates at 6000 cells/well, then treated with cyclotides, as indicated, for 72 h, then assayed using MTT. Cell viability was calculated as a percentage of control (where the control cells were treated with DMSO only). Incubations were conducted in 96-well plates in triplicate wells (technical replicates) and as three independent incubations (biological replicates, n = 3). Data are expressed as mean ± standard error of the mean (SEM). IC_50_ values were calculated using the Best Fit method in GraphPad Prism version 8.0 (IC50 Best Fit). Results are graphed in GraphPad Prism version 8 for iOS.

To evaluate the cytotoxicity of plant-derived Kalata B1 and synthetic Kalata B1, MTT assays were performed with T-98G, U-87 MG, and SH-SY5Y cell cultures. The concentrations (0–10 µM) were evaluated during a 72 h incubation period, with Best Fit IC_50_ values calculated as previously described, which indicated that each cyclotide had an IC_50_ value of <22 µM for the cell lines investigated. The natural Kalata B1 had an IC_50_ value of 5.6 µM in the SH-SY5Y cells and an IC_50_ value of 7.9 µM in the U-87 MG cell cultures. The IC_50_ values of synthetic Kalata B1 were 2.6 µM in T-98G cells and 5.4 µM in U-87 MG cells. Thus, the activity of natural Kalata B1 was comparable to synthetic Kalata B1 (Figure 7).

### 3.3. Cytotoxicity of Co-Exposure with Cyclotides and TMZ in U-87 MG and T-98G Cells

In the U-87 MG glioblastoma cells, the experimentally determined IC_50_ Best Fit value for TMZ was 1617.0 µM (Figure 5), and the IC_50_ Best Fit value for synthetic Kalata B1 was 5.4 µM (Figure 7). We were particularly interested in improving the sensitivity of glioblastoma cells to TMZ; therefore, we incubated cultures with several reduced concentrations of TMZ and synthetic Kalata B1, alone and in combination. MTT assays (as previously described) were employed to evaluate cell viability following co-exposure treatments. Cells were incubated with Kalata B1 (K) (0.5, 1.0, 2.0, and 4.0 µM) alone, TMZ (T) (100, 200, 400, and 800 µM) alone, and then co-exposed to synthetic Kalata B1 and TMZ.

**Figure 8:**
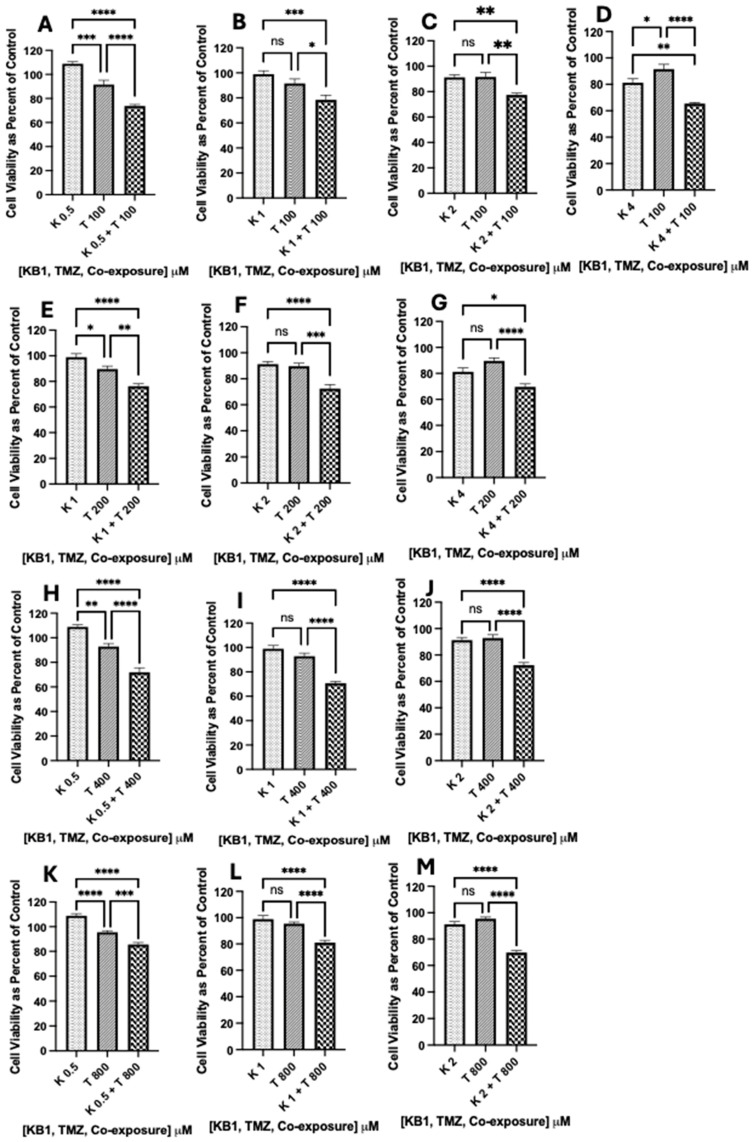
Synthetic Kalata B1 increased TMZ-induced cell death in U-87 MG glioblastoma cultures. To determine if synthetic Kalata B1 (K) enhances TMZ (T)-induced cell death in U-87 MG cells, MTT was measured after incubating the cell cultures with concentrations of K (0.5, 1.0, 2.0, and 4.0 μM) and T (100, 200, 400, and 800 μM) both alone and when co-exposed to synthetic Kalata B1 and TMZ as illustrated in panels (**A**–**M**). The first dotted bars in each panel indicate the cells exposed to concentrations of synthetic Kalata B1 alone, while the second diagonal stripe bars indicate the cells exposed to TMZ alone. The third checkered bars indicate co-exposure treatments with synthetic Kalata B1 and TMZ. The cells were plated into 96-well plates (6000 cells/well) and treated with synthetic Kalata B, as indicated, for 72 h. Cell viability was measured using MTT and expressed as a percentage of control cultures (culture medium with DMSO only). Incubations were conducted in triplicate wells (technical replicates) and as three independent incubations (biological replicates, n = 3). Data are expressed as mean ± standard error of the mean (SEM). Results of one-way analysis of variance (ANOVA) and Tukey’s multiple comparisons are graphed in GraphPad Prism version 9 for iOS with the following *p*-values: * < 0.05, ** < 0.01, *** < 0.001, and **** < 0.0001. ns = not significant.

The results of the one-way analysis of variance (ANOVA), followed by Tukey’s multiple comparisons, were graphed in GraphPad Prism version 9 for iOS and indicate that there was a significant reduction in cell viability in the following co-exposure treatments: (K 0.5 µM + T 100 µM; Figure 8A), (K 0.5 µM + T 400 µM; Figure 8H), (K 0.5 + T 800 µM; Figure 8K), (K µM 1 + T 100 µM; Figure 8B), (K 1 µM + T 200 µM; Figure 8F), (K 1 µM + T 400 µM; Figure 8I), (K 1 µM + T 800 µM; Figure 8L), (K 2 µM + T 100 µM; Figure 8C), (K 2 µM + T 200 µM; Figurer 8F), (K 2 µM + T 400 µM; Figure 8J), (K 2 µM + T 800 µM; Figure 8M), (K 4 µM + T 100 µM; Figure 8A), (K 4 µM + T 200 µM; Figure 8G). Although 3 out of the 16 co-exposure treatments analyzed (K 0.5 µM + T 200 µM, K 4 µM + T 400 µM, K µM + T 800 µM) did not significantly increase TMZ-induced cytotoxicity (Figure S5), the results confirm that 81.3% of the combination treatments analyzed provide support that synthetic Kalata B1 can chemosensitize cells to TMZ, and TMZ concentrations over 16-fold lower than the IC_50_ Best Fit value for TMZ (1615.1 µM, Figure 5B) can still result in significant cell death in U-87 MG glioblastoma cells (Figure 8). The methodology used to analyze co-exposure data are similar to a previous publication [9], and the results are consistent with co-exposure treatments of other naturally occurring cyclotides, TMZ, and glioblastoma [9].

In the T-98G glioblastoma cells, the experimentally determined IC_50_ Best Fit value for TMZ was 1160.0 µM (Figure 5), and the IC_50_ Best Fit value for synthetic Kalata B1 was 2.6 µM (Figure 7). To address our interest in chemosensitizing glioblastoma cells to TMZ, we used previously described MTT assays to analyze cell viability with T-98G cells exposed to reduced concentrations of TMZ and synthetic Kalata B1. Cells were incubated with synthetic Kalata B1 (K) (0.25, 0.5, 1.0, and 2.0 µM) alone, TMZ (T) (75, 150, 300, and 600 µM) alone, and then co-exposed to Kalata B1 and TMZ.

**Figure 9:**
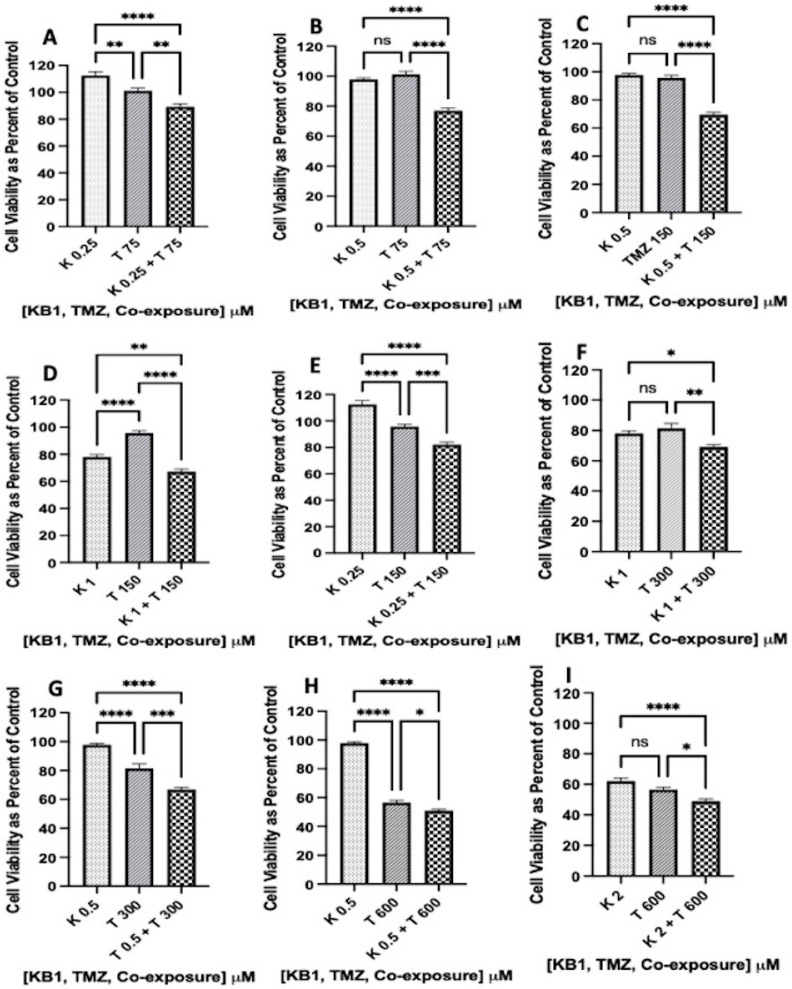
Synthetic Kalata B1 increased TMZ-induced cell death in T-98G glioblastoma cultures. To determine if Kalata B1 (K) enhances TMZ (T)-induced cell death in T-98G cells, MTT was measured after incubating cell cultures with synthetic Kalata B1 (0.25, 0.5, 1.0, and 2.0 μM) and TMZ (T 75, 150, 300, and 600 μM) both alone and when co-exposed as illustrated in panels (**A**–**I**). The first dotted bars in each panel indicate the cells exposed to synthetic Kalata B1 alone, while the second diagonal stripe bars indicate the cells exposed to TMZ alone. The third checkered bars indicate co-exposure treatments with synthetic Kalata B1 and TMZ. The cells were plated into 96-well plates (6000 cells/well) and treated with synthetic Kalata B1, as indicated, for 72 h. Cell viability was measured using MTT and expressed as a percentage of control cultures (culture medium + DMSO only). Incubations were conducted in triplicate wells (technical replicates) and as three independent incubations (biological replicates, n = 3). The data are expressed as mean ± standard error of the mean (SEM). The results of one-way analysis of variance (ANOVA) and Tukey’s multiple comparisons are graphed in GraphPad Prism version 9 for iOS, with the following *p*-values: * < 0.05, ** < 0.01, *** < 0.001, and **** < 0.0001. ns = not significant.

Results of one-way analysis of variance (ANOVA) followed by Tukey’s multiple comparisons were graphed in GraphPad Prism version 9 for iOS and indicate that there was a significant reduction in cell viability in the following co-exposure treatments: (K 0.25 + T 75 µM; Figure 9A), (K 0.25 + T 150 µM; Figure 9E), (K 0.5 + T 75 µM; Figure 9B), (K 0.5 + T 150 µM; Figure 9C), (K 0.5 + T 300 µM; Figure 9G), (K 0.5 + T 600 µM; Figure 9H), (K1 + T 150 µM; Figure 9D), (K 1 + T 300 µM; Figure 9F), and (K 2 + T 600 µM; Figure 9I). Although some treatments (K 1 µM + T 75 µM, K 22 µM + T 75 µM, K 2 µM + T 150 µM, K 0.25 + T 300 µM, K 2 µM + T 300 µM, K 1 µM + T 600 µM) did not cause significant differences in co-exposure treatments (Figure S6), at least 56.2% of the co-treatments with synthetic Kalata B1 chemosensitized T-98G glioblastoma cells to TMZ. Furthermore, the lowest co-exposure concentration (K 0.25 µM + T 75 µM; Figure 9A) used a concentration of TMZ 15-fold lower that the IC_50_ Best Fit value for TMZ in T-98G cells (1167.6 µM; Figure 5A) and still caused significantly greater cell death compared to synthetic Kalata B1 alone (0.25 µM) or TMZ alone (75 µM) (Figure 9). Low concentrations of Kalata B1, (0.5 μM in U-87 MG cells and 0.25 μM in T-98G cells) could produce a stimulatory effect, as illustrated in Figure 8 and Figure 9, when analyzed as a percent of control (no treatment). This activity has been documented in other cancer cell lines, such as human malignant glioblastoma tumor cells (U-251) and primary human umbilical vein endothelial cells (HUVEC) [30]. The analysis methods used were aligned with a previous publication [9], and results are consistent with the indication that cyclotides at various concentrations may increase TMZ toxicity in glioblastoma cell lines.

### 3.4. Phase Contrast Microscopy to Visualize Cytotoxicity in U-87 MG Cells

**Figure 10:**
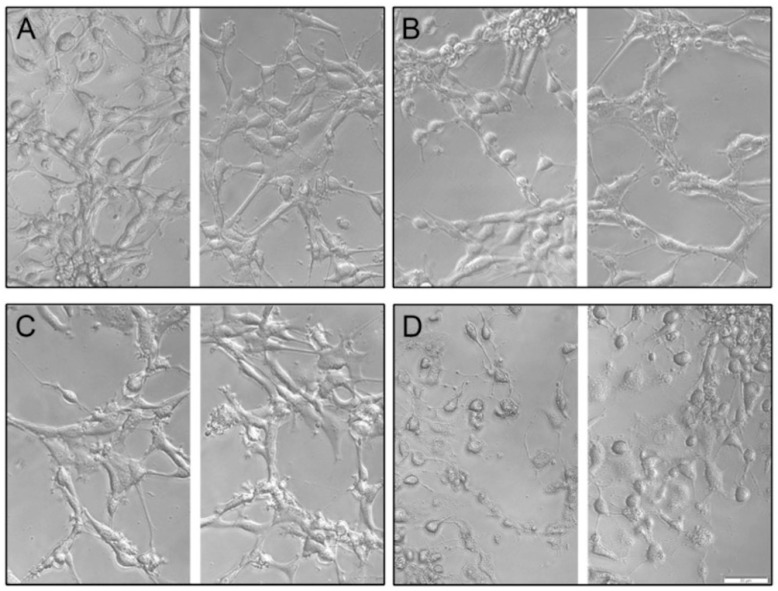
Phase contrast microscopy of U-87 GM cultures treated with Kalata B1 and temozolomide. Panels are as follows: (**A**) no treatment/control; (**B**) 150 µM temozolomide (TMZ); (**C**) synthetic Kalata B1 0.5 µM; and (**D**) 150 µM TMZ + 0.5 µM synthetic Kalata B1 for 72 h. Imaged on an Olympus IX83 inverted microscope equipped with an Olympus XM10 camera and acquired in cellSens standard. Images were adjusted for exposure in Adobe Photoshop 23.4.2 using “Levels” and cropped for consistency and to enable ease of comparison. U-87 GM cells were plated at 6000 cells/well in a 96-well plate and allowed to adhere overnight prior to treatment. Images are representative of triplicate incubations. The yellow bar in the lower right-hand corner of the image is 50 µm. 20× magnification.

U-87 MG cells play a significant role in the development of glioblastoma multiforme, and they develop resistance rapidly; therefore, we are interested in determining if differences could be seen in untreated (control) U-87 MG glioblastoma cells and those treated with TMZ (150 µM), synthetic Kalata B1 (0.5 µM), and co-treatments (150 µM TMZ + 0.5 µM synthetic Kalata B1). We performed phase contrast microscopy to visualize the changes induced by treatments that previously showed significant results in cell viability assays. In cultures incubated with 150 µM TMZ (Figure 10B) and 0.5 µM synthetic Kalata B1 (Figure 10C), little differences were observable between the control cultures (Figure 10A) and the respective treatment. This corresponded with the MTT cell viability assays, which returned almost no cell death for the U-87 MG cells treated with 0.5 µM synthetic Kalata B1, and less than 15% cell death for cells treated with ≤200 µM TMZ. Conversely, in cells undergoing co-exposure (150 µM TMZ + 0.5 µM synthetic Kalata B1), noticeable changes in microscopy images correlated with finding of cell death in Figure 8, with various shrunken, granular, blebbing cells, which suggests apoptosis (Figure 10D). Conversely, microscopy imaging of T-98G glioblastoma cells was not as clear, possibly due to variation in the pattern of growth (Figure S7).

### 3.5. Synthetic Kalata B1 Remains Stable in Human Serum

**Figure 11:**
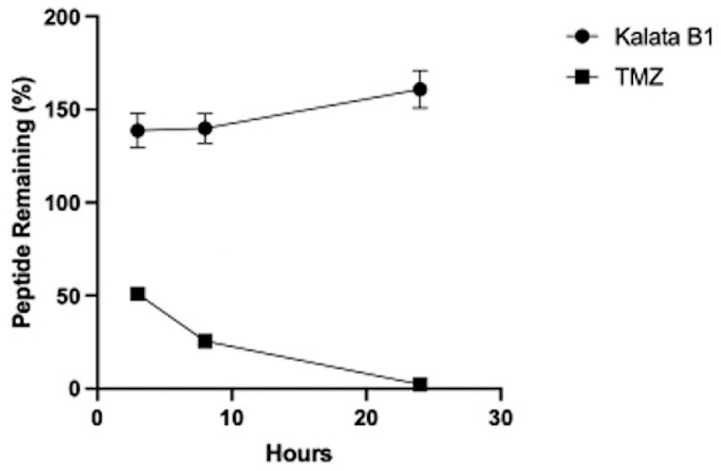
Stability of synthetic Kalata B1 and temozolomide (TMZ) in human serum over 24 h. Percentage of Kalata B1 and TMZ remaining upon incubation with human serum. The portion of serum-treated peptide remaining was determined by calculating the area of the respective elution peak in UPLC-MS and comparing it with the control without incubation. Data points are means ± SEM.

We conducted peptide stability assays in human serum isolated from male AB plasma and then analyzed with UPLC-MS. Synthetic Kalata B1 and TMZ were incubated with human serum and the portion of the peptide remaining was quantified by calculating the area of the respective elution peak in UPLC-MS, compared to the control. Figure 11 indicates that at data points of 3 h, 8 h, and 24 h, synthetic Kalata B1 remains stable, whereas the concentration of TMZ decreases to less than 50% at 3 h, less than 30% at 8 h, and <5% at 24 h.

## 4. Discussion

Glioblastoma multiforme (GBM) affects both children and adults and is the most aggressive type of cancer in the brain. Post-diagnosis, patients often survive fewer than 16 months. It is estimated that less than 7% of patients diagnosed with GBM survive longer than five years after diagnosis, in part, because resection and radiation alone are not efficacious, and many patients become resistant to currently approved FDA drugs [31,32]. In this study, we investigated the effects of cyclotides on the U-87 MG and T-98G glioblastoma cell lines to determine if they can chemosensitize glioblastoma cell lines to the FDA-approved drug temozolomide (TMZ). TMZ does offer some hope to patients as a chemotherapeutic agent; however, roughly 50% of patients do not respond to TMZ, and those patients in which TMZ is initially efficacious often develop rapid drug resistance [33]. Therefore, studies to discover adjuvant methods to treat GBM are vital.

To this end, we investigated several naturally occurring cyclotides (CyO3, CyO19, Kalata B1, Kalata B7, Vitri E) and one synthetic cyclotide, Kalata B1. The plant-derived compounds are commonly found in *Viola odorata* L. and *Viola tricolor* L., two species of violets in the family Violaceae. Although the cytotoxicity of Kalata B1 has been reported in U-87 and U-251 glioblastoma cells using an MTT assay [30], different experimental conditions, incubation periods, and dyes for assessing cell viability were used in the current study, yet the results were still comparable. Tang and colleagues [34] evaluated the cell viability of several cyclotides in a sulforhodamine B (SRB) assay, and several of them such as Cycloviolacin O2, Vitri A, and Vitri F displayed toxicity towards U-251 glioblastoma cells that was equipotent to that of the U-87 MG and T-98G cellular data provided in the current research (Table 4).

Cyclotides isolated from *Viola sumatrana* Miq. also lead to cytotoxicity in MTT assays, with CyO2 displaying more potent activity (IC_50_ = 0.45 and 0.79 µM) compared to Kalata B1 (IC_50_ = 3.21 and 10.88 µM) in the U-87 and U-251 glioblastoma cells, respectively [35]. Our previous research also indicates that cyclotides such as CyO2 (IC_50_ = 4.5 µM), CyO13 (IC_50_ = 2.57 µM), and Varv peptide A (IC_50_ = 2.91 µM) display cytotoxicity in a range similar to the data presented in this research (IC_50_ = 2.4–21.1 µM) (Figure 6). This indicates that cyclotides alone may be promising compounds for further investigation in GBM chemotherapy. The five cyclotides in this research displayed dose-dependent cytotoxicity in human neuroblastoma (SH-SY5Y) and human glioblastoma (U-87 MG, and T-98G) cells (Table 4). Previous studies indicate that the cytotoxic potency of cyclotides can be significantly affected by substitutions or removal of amino acids in the cyclotide sequences. The most striking evidence of this fact was a 48-fold decrease in cytotoxicity in lymphoma cells that Lindholm and colleagues [36] observed when the conserved glutamic acid (E) was removed from cyclotides. The extent of surface exposure of hydrophobic patches in cyclotides has been known to affect bioactivity, which indicates that structure plays an important role in bioactivity. In the present research, two bracelet cyclotides (CyO3 and CyO19) and three Möbius cyclotides (Kalata B1, Kalata B7, and Vitri E) were screened. CyO3 and CyO19 have varying amino acids in loop 3 and loop 6, while Kalata B1, Kalata B7, and Vitri E have a distinct complement of amino acids in loops 3, 5, and 6. Despite these differences, all the cyclotides in this research displayed IC_50_ values of <22 µM.

Challenges commonly associated with treating cancer can include low drug uptake across the cellular lipid bilayer, the activity of pumps, such as p-glycoprotein, that efflux drugs out of the cell, and the impenetrability of the blood–brain barrier [11,37]. Our previous research provided supporting evidence that cyclotides such as CyO2 can act as adjuvants in drug-resistant breast cancer cells, whereby cyclotides sensitize human breast adenocarcinoma (MCF-7) and drug-resistant human breast adenocarcinoma (MCF-7/ADR) cells to the FDA-approved anti-cancer drug, doxorubicin. In that case, one mechanism of cytotoxicity was most likely the development of tetramer pores on the plasma membrane of the cancer cell, which allowed for the passage and uptake of doxorubicin using the SYTOX green assay. Interestingly, Cycloviolacin O2 did not produce significant membrane disruption in primary human brain endothelial cells [11]. The literature indicates that the bioactivity of Kalata B1 has yet to be determined in noncancerous human brain cells; therefore, another goal of our future research will include an evaluation of Kalata B1 in unaffected brain cells.

Although a mechanistic evaluation of Kalata B1 bioactivity was beyond the scope of this research, a brief discussion of the previous mechanistic studies is justified. For instance, Henriques and colleagues [35] provided supporting evidence that cyclotides can target phosphatidylethanolamine (PE)-enriched phospholipids and penetrate the cell via both endocytosis and membrane translocation. An additional study indicated that at least one mechanism of activity of Kalata B1 and Kalata B2 is consistent with the formation of lipidic toroidal pores that decrease the critical packing parameter and act much like a surfactant [38]. Furthermore, bioactivity studies for immune-related disorders have investigated additional mechanisms of bioactivity, with investigations documenting that a Kalata B1 mutant, named [T20k] Kalata B1, could decrease the expression of interleukin-2 (IL-2), IL-2 cytokine secretion, and IL-2 gene expression [39]. Since these reports indicate the mechanisms of cyclotide bioactivity are complex, future evaluations with synthetic Kalata B1 and any analogs should also focus on delineating their specific mechanisms of action regarding GBM.

Here, one aim of our experiments was to assess if co-exposure of TMZ with synthetic Kalata B1 could increase the sensitivity of U-87 MG and T-98G cells to TMZ-induced cytotoxicity. The results lay the foundation for transitioning towards an in vivo xenograft mouse model to begin determining whether a combination approach with cyclotides and TMZ may reduce the required effective dose of TMZ and perhaps decrease the development of TMZ resistance in glioblastoma cells. To be considered potentially efficacious, the combination treatment must be significantly different from that of synthetic Kalata B1 alone or TMZ alone, which was indicated in the co-exposure treatment in U-87 MG (Figure 8) and T-98G (Figure 9) glioblastoma cell lines. Although obtaining the IC_50_ values of combination treatments was not a primary objective of this research, future studies will focus on obtaining full dose response curves for each agent (synthetic Kalata B1 and TMZ), as well as fixed ratio (synthetic Kalata B1 + TMZ) treatments followed by an analysis of the combination index to determine if the activity is synergistic, additive, or antagonistic.

The development of an in vivo mouse model is vital, yet it requires expertise, significant finances, and large quantities of the target cyclotide. To this end, an early investigation with the cyclotide Cycloviolacin O2 was completed, demonstrating that it had an abrupt in vivo toxicity profile, since a single injection at 2 mg/kg was lethal, yet there were no signs of discomfort in mice treated with 1.5 mg/kg in tumor mouse models [40]. A mouse model of anaplastic large cell lymphoma (ALCL) was developed, and the Kalata B1 mutant, named [T20K] Kalata B1, decreased tumor weight and increased apoptosis, which led researchers to assert that cyclotide-based drugs may be a promising therapy for patients with ALCL [41]. A full report on the safety and efficacy of synthetic Kalata B1 has not been conducted in mouse models of GBM; therefore, one of our future objectives is the development of an in vivo GBM mouse model.

Our previous research indicates that cyclotides (CyO2 and Varv peptide A) chemosensitize glioblastoma cells to TMZ. In fact, the data indicated that 8-fold less TMZ was required to cause significant cell death in U-87 MG cells exposed to CyO2 co-exposure with TMZ. Likewise, 2-fold less TMZ was needed for significant cytotoxicity in U-87 MG cells co-exposed to Varv peptide A. The data presented here indicate that the chemosensitizing effects of synthetic Kalata B1 are comparable to the activity illustrated with CyO2 and Varv peptide A [9]. Therefore, we provide supporting evidence that synthetic Kalata B1 increased TMZ-induced cell death in both U-87 MG and T-98G glioblastoma cells (Figure 8 and Figure 9). Although MTT assays provide valuable information regarding cell viability, it is important to fully evaluate the effects of synthetic Kalata B1 on TMZ for GBM treatment; therefore, future priorities include investigating the cyclotide’s activity in other bioactivity assays, such as the lactate dehydrogenase (LDH) assay, the colony formation assay, and the blood–brain barrier assay.

Symptoms of GBM include seizures, headaches, nausea, and memory loss, and the side effects of TMZ can not only intensify headaches and nausea but also include diarrhea, constipation, loss of appetite, and sores in the mouth and throat. TMZ is an oral DNA alkylating agent that induces cell cycle arrest at G2/M, and its cytotoxicity is mediated by the addition of methyl groups at N^7^ and O^6^ sites on guanines and the O^3^ site on adenines in genomic DNA. A thymine instead of a cytosine is inserted during the alkylation of the O^6^ site on guanine, which can cause cell death during DNA replication. Patient resistance to TMZ is a significant challenge in TMZ chemotherapy. The mechanism of TMZ resistance involves methylated sites that can remain mutated, be fixed via DNA mismatch repair (MMR), be dealkylated by the action of a demethylating enzyme including O^6^-methylguanine methyltransferase (MGMT), or be removed by base excision repair (BER) by the activity of DNA glycosylase, such as alkylpurine-DNA-N-glycosylase (APNG). When MMR is expressed, human glioblastoma cells are TMZ-sensitive, and when MGMT, BER, and APNG proteins are expressed, then the cells develop resistance to TMZ [31]. In the current research, the IC_50_ value of the TMZ-resistant cell line T-98G (1160.0 μM) is within the range of the IC_50_ values (>250–1585 μM) summarized by Lee [31]. Interestingly, the IC_50_ value of the report TMZ-sensitive U-87 MG cell line (1617.0 μM) is higher than the range (7 to less than 500 μM) reviewed. Human glioblastoma cells can acquire resistance rapidly or over time; therefore, a speculative explanation for the higher IC_50_ value of the U-87 MG cell line in this study is that these cells may have been expressing, singularly or in combination, MGMT, BER, and APNG proteins.

One of our objectives is to promote research that investigates methods to decrease the required concentrations of drugs such as TMZ for efficacy in vivo. Therefore, it is important to analyze the stability of cyclotides in human serum. The trypsin inhibitor cyclotide, MCoTI-II, has been extracted from the seeds of *Momordica cochinchinensis* Spreng. [Cucurbitaceae]. Chan and colleagues [27] performed serum stability assays and documented that MCoTI-II remained stable in human serum for 24 h with 100% peptide remaining. Huang and colleagues [28] repeated these experiments with MCoTI-II and an analog, MCo-RMI. Both peptides remained stable in human serum for at least 24 h. We adopted their methodology and herein provide evidence that synthetic Kalata B1 also remains stable in human serum isolated from male AB plasma for at least 24 h.

*Momordica cochinchinensis* is a perennial melon that expresses the trypsin inhibitor MCoTI-II, and it is a cell-penetrating peptide, which is a group of peptides reported to cross the blood–brain barrier. Wang and colleagues [42] compared the four-disulfide peptide (chlorotoxin) to the three-disulfide peptide (MCoTI-II) and demonstrated that chlorotoxin did cross the blood–brain barrier in a mouse model, yet MCoTI-II was not translocated across the brain. Another report of a radio-labelled analog modified from another trypsin-inhibitor cyclotide, MCoTI-I, extracted from *M. cochinchinensis*, indicated that the analog ([^64^CU] MCo-CVX-6D) increased the uptake of U-87-stb-C-X-C chemokine receptor type 4 (CXCR4)-expressing cells in vitro and in vivo [43]. MCoTI-I and MCoTI-II are characterized as cyclotides in the trypsin-inhibitor subfamily; however, they have also been regarded as cyclic knottins because they contain a cystine knot motif as with all other cyclotides, but they have no further sequence similarity with the bracelet and Möbius subfamilies of cyclotides [43,44,45]. Therefore, future directives include optimizing a PAMPA blood–brain barrier (BBB) model to quantify the passage of synthetic Kalata B1 (a cyclotide in the Möbius subfamily) across a simulated BBB and developing a xenograft mouse model of GBM to study cyclotide bioactivity in vivo.

## 5. Conclusions

In conclusion, this research identified cyclotides with techniques including UPLC-PDA and HPLC-UV and provided sequence confirmation for synthetic Kalata B1 using orbitrap LC-MS, followed by structural confirmation with NMR spectrometry. Human stability assays verified that synthetic Kalata B1 remains stable in serum for at least 24 h, and the evaluation of bioactivity in human glioblastoma cell lines (U-87 MG and T-98G) indicate that the cyclotides, Cycloviolacin O3, Cycloviolacin O19, natural Kalata B1, synthetic Kalata B1, and Vitri E cause dose-dependent cytotoxicity. Co-exposure experiments with synthetic Kalata B1 and TMZ provided initial proof-of-concept that the synthetic Kalata B1 may enhance TMZ toxicity in U-87 MG and T-98G glioblastoma cells. Therefore, continued research on the development of an adjuvant method towards the treatment of GBM with cyclotides and TMZ is warranted.

## Figures and Tables

**Table 1:** MS/MS analysis of peptide 1 using orbitrap mass spectrometry.

Sequence	*m*/*z*	Observed Mass Difference	Expected Mass Difference
N	845.3799		
G	731.33765	114.04236	114.04293
L	674.31616	57.02148	57.02146
P	561.23236	113.0838	113.08406
V	464.17984	97.05252	97.05276
C	365.111172	99.06812	99.06841
G	205.0815	160.03023	160.03059
E	148.06015	57.02135	57.02146
			148.060984

**Table 2:** MS/MS analysis of peptide 2 using orbitrap mass spectrometry.

Sequence	*m*/*z*	Observed Mass Difference	Expected Mass Difference
R			175.119501
T	175.1182	101.04756	101.04768
C	276.16638	160.03055	160.03059
V	436.19693	99.06827	99.06841
P	535.2652	97.05267	97.05276
W	632.31787	186.07916	186.07931
S	818.39703	87.03192	87.03203
C	905.42896	160.03052	160.03059
T	1065.45947	101.04785	101.04768
C	1166.50732	160.03113	160.03059
G	1326.53845	57.02136	57.02146
P	1383.55981	97.05212	97.05276
T	1480.61194	101.04785	101.04768
N	1581.65979	114.04272	114.04293
C	1695.70251	160.03076	160.03059
T	1855.73328	57.02209	57.02146
G	1956.7821	57.02124	57.02146
G	2013.8042	99.06763	99.06841
V	2070.82544	ND	
C	2169.89307	ND	
T	ND		
	ND		

ND, not detected.

**Table 3:** Chemical shifts of Kalata B1.

Residues							
	HN	Hα	Hβ	Hγ	Hδ	Others	3JNH-CaH (Hz)
Gly1	8.66	4.15, 3.50	-	-	-	-	-
Leu 2	7.67	4.96	1.84, 1.39	1.62	0.89, 0.82	-	10.13
Pro 3	-	4.98	2.37, 1.63	2.06, 1.96	3.70, 3.67	-	-
Val 4	8.04	4.57	2.50	0.79, 0.75	-	-	9.5
Cys 5	7.90	4.38	3.26, 2.91	-	-	-	8.83
Gly 6	8.43	3.66	-	-	-	-	-
Glu 7	7.08	4.71	1.93, 1.78	2.45	-	-	9.11
Thr 8	8.36	4.47	4.36	1.05	-	-	9.25
Cys 9	8.25	4.85	3.10, 2.83	-	-	-	11.2
Val 10	8.43	3.76	1.95	0.94, 0.86	-	-	9.2
Gly 11	8.59	4.17, 3.75	-	-	-	-	-
Gly 12	8.13	4.32, 3.95	-	-	-	-	-
Thr 13	7.76	4.62	4.01	1.06	-	-	-
Cys 14	8.60	4.61	2.95, 2.68	-	-	-	8.77
Asn 15	9.33	4.61	2.94, 2.70	-	-	-	9.98
Thr 16	8.27	4.41	4.12	1.24	-	-	9.36
Pro 17	-	4.18	2.23, 1.82	2.06, 1.92	4.06, 3.62	-	-
Gly 18	8.68	4.19, 3.60	-	-	-	-	-
Cys 19	7.60	5.23	3.73, 2.53	-	-	-	10.28
Thr 20	9.42	4.44	3.95	1.04	-	-	9.17
Cys 21	8.85	4.5	3.03, 2.73	-	-	-	9.62
Ser 22	8.87	4.68	3.76	-	-	-	9.62
Trp 23	7.88	3.98	3.16	-	7.21	10.31 (HNε), 7.34 (Hε3), 7.13 (HH2)	-
						7.42 (HZ2), 7.02 (HZ3)	-
Pro 24	-	3.33	1.59 -0.30	1.27, 1.19	3.15, 3.11	-	-
Val 25	8.18	4.12	1.83	0.76, 0.73	-	-	9.01
Cys 26	7.63	5.02	3.13, 2.66	-	-	-	8.37
Thr 27	9.77	4.98	3.62	0.79	-	-	9.74
Arg 28	8.62	4.67	1.58	1.34	3.07	6.86 (Hε)	10.6
Asn 29	9.45	4.31	3.00, 2.75	-		-	9.87

**Table 4:** Toxicity and IC_50_ values of five cyclotides and TMZ in SH-SY5Y, U-87 MG, and T-98G cell cultures.

Cell Type	Compound	IC_50_ (μM) ± S.D.
U-87 MG	CyO3	2.6 ± 0.05
	CyO19	2.4 ± 0.07
	Kalata B1 ^a^	7.9 ± 1.66
	Kalata B1 (synthetic)	5.4 ± 1.06
	Kalata B7	10.1 ± 1.74
	Vitri E	21.1 ± 2.90
	TMZ ^a^	2898.0 ± 6.00
	TMZ	1617.0 ± 5.56
T-98G	Kalata B1 (synthetic)	2.6 ± 0.24
	TMZ	1160.0 ± 5.31
SH-SY5Y	Kalata B1 ^a^	5.6 ± 1.71
	TMZ ^a^	393.0 ± 4.50

^a^ indicates previously published data [9].

## Data Availability

Data are contained within the article and in the Supplementary Materials. Any additional data presented in this study are available on request from the corresponding authors.

## Supplementary Materials

The following supporting information can be downloaded at: https://www.mdpi.com/article/10.3390/biomedicines12102216/s1, Figure S1: Verification of synthetic Kalata B1 purity from CSBio (Menlo Park, CA, USA); Figure S2: The structural feature of Kalata B1; Figure S3: The folding of Kalata B1 from a random coil structure to cyclic structure using NOE distance constraints. Figure S4: Synthetic and natural Kalata B1; Figure S5: synthetic Kalata B1 did not significantly increase TMZ-induced cytotoxicity in U-87 MG glioblastoma cell culture when using the following co-exposure concentrations: Kalata B1 (K) 0.5 µM + Temozolomide (T) 200 µM, K 4 µM + T 400 µM, and K 4 µM + T 800 µM; Figure S6: Synthetic Kalata B1 did not significantly increase TMZ-induced cytotoxicity in T-98G glioblastoma cell culture when using the following co-exposure concentrations: Kalata B1 (K) 1 µM + Temozolomide 75 µM, K 2 µM + T 75 µM, K 2 µM + T 150 µM, K 0.25 + T 300 µM, K 2 µM + T 300 µM, K 1 µM + T 600 µM; Figure S7: Light microscopy of T-98G cultures treated with Kalata B1 and temozolomide.

## References

[1] Gran L., Sandberg K., Sletten K. (2000). A plant containing uteroactive peptides used in African traditional medicine. J. Ethnopharmacol..

[2] Abdul Ghani H., Henriques S.T., Huang Y.H., Swedberg J.E., Schroeder C.I., Craik D.J. (2017). Structural and functional characterization of chimeric cyclotides from the Möbius and trypsin inhibitor subfamilies. Biopolymers.

[3] Jennings C., West J., Waine C., Craik D., Anderson M. (2001). Biosynthesis and insecticidal properties of plant cyclotides: The cyclic knotted proteins from *Oldenlandia affinis*. Proc. Natl. Acad. Sci. USA.

[4] Gruber C.W., Čemažar M., Anderson M.A., Craik D.J. (2007). Insecticidal plant cyclotides and related cystine knot toxins. Toxicon.

[5] Strömstedt A.A., Park S., Burman R., Göransson U. (2017). Bactericidal activity of cyclotides where phosphatidylethanolamine-lipid selectivity determines antimicrobial spectra. Biochim. Biophys. Acta Biomembr..

[6] Slazak B., Kapusta M., Strömstedt A.A., Słomka A., Krychowiak M., Shariatgorji M., Andrén P.E., Bohdanowicz J., Kuta E., Göransson U. (2018). How Does the Sweet Violet (*Viola odorata* L.) Fight Pathogens and Pests—Cyclotides as a Comprehensive Plant Host Defense System. Front. Plant Sci..

[7] Henriques S.T., Craik D.J. (2010). Cyclotides as templates in drug design. Drug Discov. Today.

[8] Azmi S., Mustafa M., Shoaib S., Hussain M.K. (2022). Structures, Functions and Therapeutic Potential of Cyclotides. J. Explor. Res. Pharm..

[9] Gerlach S.L., Dunlop R.A., Metcalf J.S., Banack S.A., Cox P.A. (2022). Cyclotides Chemosensitize Glioblastoma Cells to Temozolomide. J. Nat. Prod..

[10] Ireland D.C., Wang C.K., Wilson J.A., Gustafson K.R., Craik D.J. (2008). Cyclotides as natural anti-HIV agents. Biopolymers.

[11] Gerlach S.L., Rathinakumar R., Chakravarty G., Göransson U., Wimley W.C., Darwin S.P., Mondal D. (2010). Anticancer and chemosensitizing abilities of cycloviolacin 02 from Viola odorata and psyle cyclotides from *Psychotria leptothyrsa*. Biopolymers.

[12] Gerlach S.L., Chandra P.K., Gunasekera S., Göransson u Wimley W.C., Braun S.E., Mondal D. (2019). The membrane-active phytopeptide cycloviolacin O_2_ targets HIV-1 infected cells and infectious viral particles to potentiate the efficacy of antiretroviral drugs. Medicines.

[13] Janjua T.I., Rewatkar P., Ahmed-Cox A., Saeed I., Mansfeld F.M., Kulshreshtha R., Kumeria T., Ziegler D.S., Kavallaris M., Mazzieri R. (2021). Frontiers in the treatment of glioblastoma: Past, present, and emerging. Adv. Drug Deliv. Rev..

[14] Wu W., Klockow J.L., Zhang M., Lafortune F., Chang E., Jin L., Wu Y., Daldrup-Link H.E. (2021). Glioblastoma multiforme (GBM): An overview of current therapies and mechanisms of resistance. Pharmacol. Res..

[15] Stupp R., Mason W.P., van den Bent M.J., Weller M., Fisher B., Taphoorn M.J.B., Belanger K., Brandes A.A., Marosi C., Bogdahn U. (2005). Radiotherapy plus concomitant and adjuvant temozolomide for glioblastoma. N. Engl. J. Med..

[16] Singh N., Miner A., Hennis L., Mittal S. (2021). Mechanisms of temozolomide resistance in glioblastoma—A comprehensive review. Cancer Drug Resist..

[17] Rajendran S., Slazak B., Mohotti S., Muhammad T., Strömstedt A.A., Kapusta M.M., Wilmowicz E., Göransson U., Hettiarachchi C.M., Gunasekera S. (2023). Screening for Cyclotides in Sri Lankan Medicinal Plants: Discovery, Characterization, and Bioactivity Screening of Cyclotides from *Geophila repens*. J. Nat. Prod..

[18] Rance M.O., Sørensen G., Bodenhausen G., Wagner R.R., Wüthrich K. (1983). Improved spectral resolution in COSY 1H NMR spectra of proteins via double quantum filtering. Biochem. Biophys. Res. Commun..

[19] Rucker S.P., Shaka A. (1989). Broadband homonuclear cross polarization in 2D NMR using DIPSI-2. Mol. Phys..

[20] Bothner-By A.A., Stephens R., Lee J., Warren C.D., Jeanloz R. (1984). Structure determination of a tetrasaccharide: Transient nuclear Overhauser effects in the rotating frame. J. Am. Chem. Soc..

[21] Bax A., Davis D.G. (1985). Practical aspects of two-dimensional transverse NOE spectroscopy. J. Mag. Reson..

[22] Kumar A., Ernes R., Wüthrich K. (1980). A two-dimensional nuclear Overhauser enhancement (2D NOE) experiment for the elucidation of complete proton-proton cross-relaxation networks in biological macromolecules. Biochem. Biophys. Res. Commun..

[23] Goddard T.D., Kneller D.G. (2000). SPARKY 3.

[24] Lee W., Tonelli M., Markley J.L. (2015). NMRFAM-SPARKY: Enhancement software for biomolecular NMR spectroscopy. Bioinformatics.

[25] Guntert P.A. (2004). Automated NMR structure calculation with CYANA. Methods Mol. Biol..

[26] Mosmann T. (1983). Rapid colorimetric assay for cellular growth and survival: Application to proliferation and cytotoxicity assays. J. Immunol. Methods.

[27] Chan L.Y., Gunasekera S., Henriques S.T., Worth N.F., Le S.-J., Clark R.J., Campbell J.H., Craik D.J., Daly M.L. (2011). Engineering pro-angiogenic peptides using stable, disulfide-rich cyclic scaffolds. Blood..

[28] Huang Y.H., Chaousis S., Cheneval O., Craik D.J., Henriques S.T. (2015). Optimization of the cyclotide framework to improve cell penetration properties. Front. Pharmacol..

[29] Wüthrich K. (1986). NMR of Proteins and Nucleic Acids.

[30] Niyomploy P., Chan L.Y., Poth A.G., Colgrave M.L., Sangvanich P., Craik D.J. (2016). Discovery, isolation, and structural characterization of cyclotides from *Viola sumatrana* Miq. Biopolymers.

[31] Young R.M., Jamshidi A., Davis G., Sherman J.H. (2015). Current trends in the surgical management and treatment of adult glioblastoma. Ann. Transl. Med..

[32] Lee S.Y. (2016). Temozolomide resistance in glioblastoma multiforme. Genes Dis..

[33] Bi W.L., Beroukhim R. (2014). Beating the odds: Extreme long-term survival with glioblastoma. Neuro-Oncology.

[34] Tang J., Wang C.K., Pan X., Yan H., Zeng G., Xu W., He W., Daly N.L., Craik D.J., Tan N. (2010). Isolation and characterization of cytotoxic cyclotides from Viola tricolor. Peptides.

[35] Henriques S.T., Huang Y.H., Chaousis S., Sani M.-A., Poth A.G., Separovic F., Craik D.J. (2015). The Prototypic Cyclotide Kalata B1 Has a Unique Mechanism of Entering Cells. Chem. Biol..

[36] Lindholm P., Göransson U., Johansson S., Claeson P., Gullbo J., Larsson R., Bohlin L., Backlund A. (2002). Cyclotides: A novel type of cytotoxic agents. Mol. Cancer Ther..

[37] Hall K., Lee T.H., Daly N.L., Craik D.J., Aguilar M.I. (2012). Gly (6) of kalata B1 is critical for the selective binding to phosphatidylethanolamine membranes. Biochim. Biophys. Acta.

[38] Cransfield C.G., Henriques S.T., Martinac B., Duckworth P., Craik D.J., Cornell B. (2017). Kalata B1 and Kalata B2 have a surfactant-like activity in phosphatidylethanolamine-containing lipid membranes. Langmuir.

[39] Gründemann C., Thell K., Lengen K., Garcia-Käufer M., Huang Y.-H., Huber R., Craik D.J., Schabbauer G., Gruber C.W. (2013). Cyclotides suppress human T-lymphocyte proliferation by an interleukin 2-dependent mechanism. PLoS ONE.

[40] Burman S., Svedlund F., Felth J., Hassan S., Herrmann A., Clark R.J., Craik D.J., Bohlin L., Claeson P., Göransson U. (2010). Evaluation of toxicity and antitumor activity of cycloviolacin O_2_ in mice. Biopolymers.

[41] Lind J., Hellinger R., Kudweis P., Moll H.P., Gattringer J., Thell K., Edtmayer S., Gruber C.W., Stoiber D., Kollmann K. (2022). The nature inspired peptide [T20k]-kalata B1 induces anti-tumor effects in anaplastic large cell lymphoma. Biomed. Pharmacother..

[42] Wang C.K., Stalmans S., De Spiegeleer B., Craik D.J. (2016). Biodistribution of the cyclotide MCoTI-II, a cyclic disulfide-rich peptide drug scaffold. J. Pept. Sci..

[43] Hernandez J., Gagnon J., Chiche L., Nguyen T., Andrieu J., Heitz A., Hong T.T., Pham T., Nguyen D.L. (2002). Squash trypsin inhibitors from Momordica cochinchinensis exhibit an atypical macrocyclic structure. Biochemistry.

[44] Lesniak W.G., Aboye T., Chatterjee S., Camarero J.A., Nimmagadda S. (2017). In vivo Evaluation of an Engineered Cyclotide as Specific CXCR4 Imaging Reagent. Chemistry.

[45] Felizmenio-Quimio M.E., Daly N.L., Craik D.J. (2001). Circular proteins in plants: Solution structure of a novel macrocyclic trypsin inhibitor from Mormordica cochinchinensis. J. Biol. Chem..

