# Supplementary material for: Kalata B1 Enhances Temozolomide Toxicity to Glioblastoma Cells
Source: Biomedicines. 2024 Sep 28;12(10):2216. doi: 10.3390/biomedicines12102216 (PMC11505038; doi:10.3390/biomedicines12102216)
Supplement: Supplementary file 1 [file biomedicines-12-02216-s001.zip › biomedicines-3060119-supplementary.pdf]

Supplementary Materials: The following supporting information can be downloaded at:

Quality Control Record

Product: Kalata-B1

Sequence: Cyclo(Gly-Leu-Pro-Val-Cys-Gly-Glu-Thr-Cys-Val-Gly-Gly-Thr-Cys-Asn-Thr-Pro-Gly-Cys-Thr-Cys-Ser-Trp-Pro-Val-Cys-Thr-Arg-Asn)

Note: Lactam Bond, head to tail; Natural Oxidation

Product No.: CS9566 Expected M.W.: 2892.35 Found M.W.: 2891.60 Lot: X477

APPEARANCE: White Powder

MOLECULAR WEIGHT VERIFICATION: Confirmed

PURITY: Instrument: Agilent 1290 97.51%

Condition: HPLC column in TFA System

Gradient: 30-60% Buffer B in 20 minutes

Buffer A: 0.1% TFA in H_2O

Buffer B: 0.1% TFA in ACN

Wavelength: 214 nm

Column: Phenomenex Luna C18 5μm 100Å, 4.6 x 250 mm

ELLMAN'S TEST: Complies

SUGGESTIONS FOR PEPTIDE DISSOLUTION: 0.1% TFA in Water

COUNTERIONS PRESENT: TFA Salt

STORAGE: All peptides should be stored dry at -20°C

This material is NOT FOR HUMAN USE. This material is not listed as hazardous by •NIOSH/RTECS. Therefore, no SAFETY DATA SHEET is required. However, the chemical, physical and toxicological properties of this product have not been thoroughly investigated. Therefore, please exercise due care when handling this material. This action is in compliance with State and Federal OSHA standards and regulations.

Quality Control:

Date: May 13, 2022

Sample Name: CS9566
Lot# X477
Instrument 1 Agilent 1290
Instrument ID: E195
Injection Date: 5/10/2022
Inj. Volume: 5.0 uL

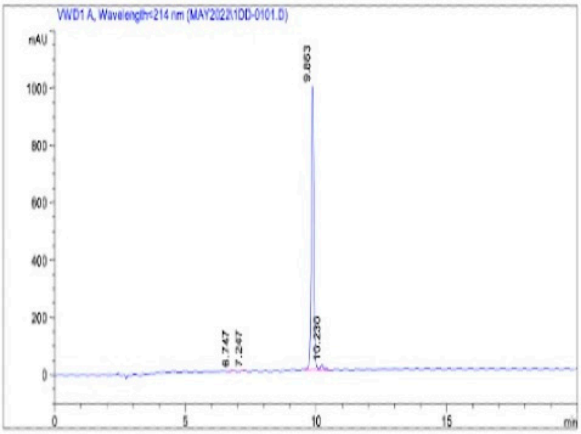

Data file name: C:\CHEM32\1\DATA\MAY2022\1DD-0101.D
Acq. Method: C:\Chem32\1\DATA\MAY2022\QC 2022-05-10 16-13-57\30-60-20-1-2.M

Column: Phenomenex Luna C18 (2) 5u 100A, 250x4.6mm
Buffer A: 0.1% TFA in H2O
Buffer B: 0.1% TFA in ACN
Wavelength: 214 nm
Flow Rate: 1 ml/minute
Column Temperature: 25C
Gradient: 30-60% B in 20 minutes

Peak #	RT [min]	Area	Height	Area %
1	6.747	16.54	2.83	0.24
2	7.247	10.73	2.02	0.16
3	9.863	6653.89	988.93	97.51
4	10.230	142.37	16.10	2.09

Figure S1. Verification of synthetic Kalata B1 purity from CSBio (Menlo Park, CA, USA).

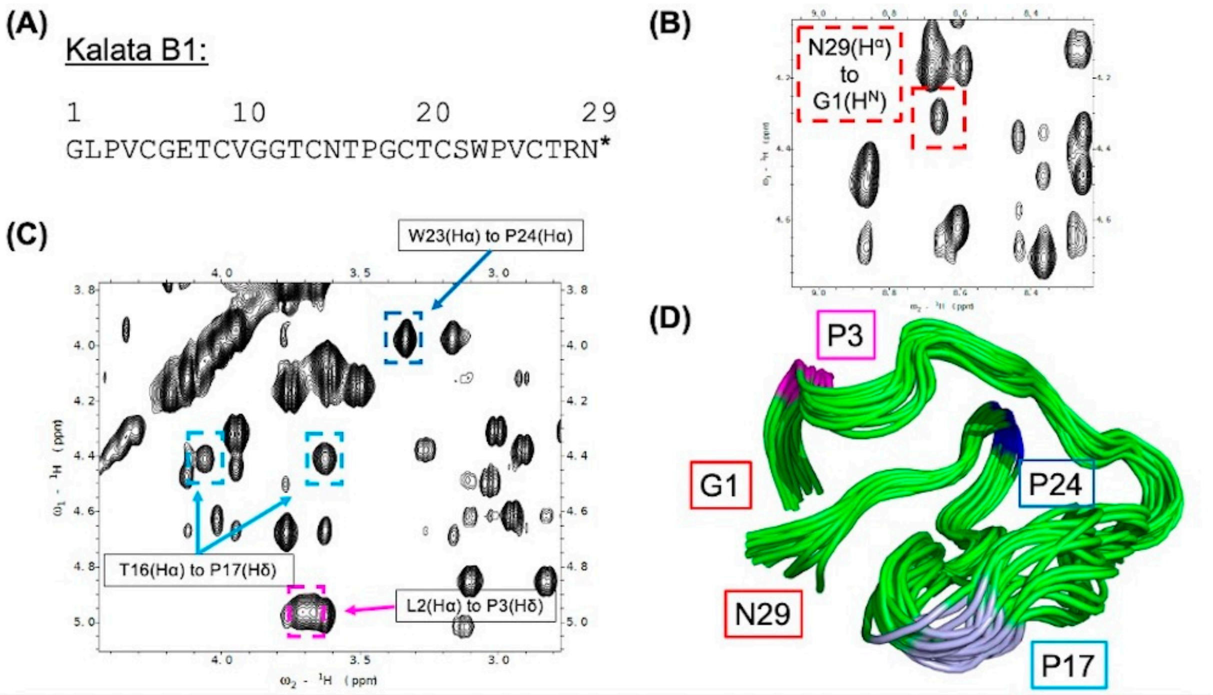

Figure S2: The structural features of Kalata B1. (A) The amino acid sequence of Kalata B1 with the * indicates that N29 is connected to G1 as a cyclic peptide. (B) The 2D NOESY spectrum indicates the sequential NOE cross peaks of Asn 29 to Gly 1 (red dashed box). (C) The 2D NOESY spectrum indicates the sequential NOE cross peaks of Pro 3 (dashed magenta box), Pro 17 (dashed light blue box), and Pro 24 (dashed blue box). (D) The ensemble with 20 structures of Kalata B1 displays the peptide backbone. Gly 1 (red box), Asn 29 (red box), Pro 3 (magenta box), Pro 17 (light blue box), and Pro 24 (blue box) are noted on the structures.

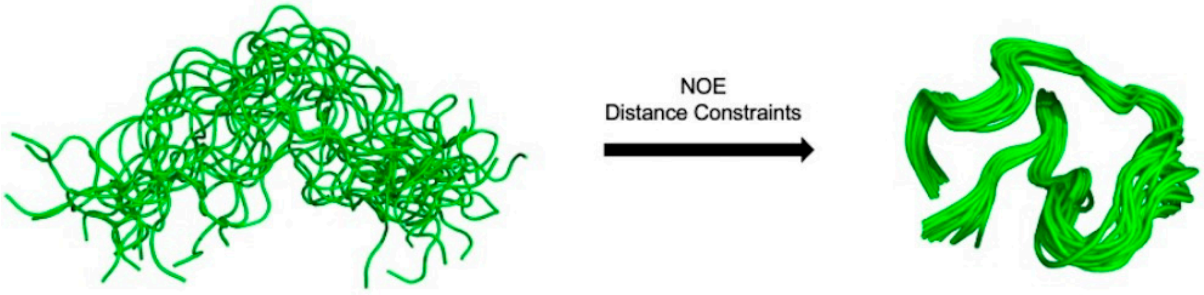

Figure S3: The folding of Kalata B1 from a random coil structure to the cyclic structure using NOE distance constraints.

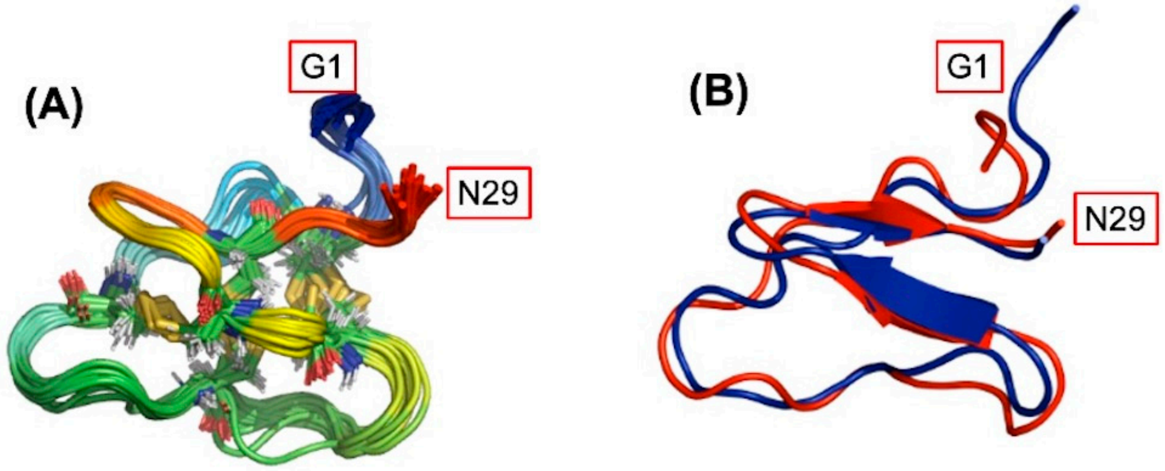

Figure S4: Synthetic and Natural Kalata B1 (A) Ensemble of 20 lowest energy structures of Kalata B1. (B) Three-dimensional alignment of the structure of the synthetic Kalata B1 (red) and the naturally derived peptide (blue, PDB: 2khb).

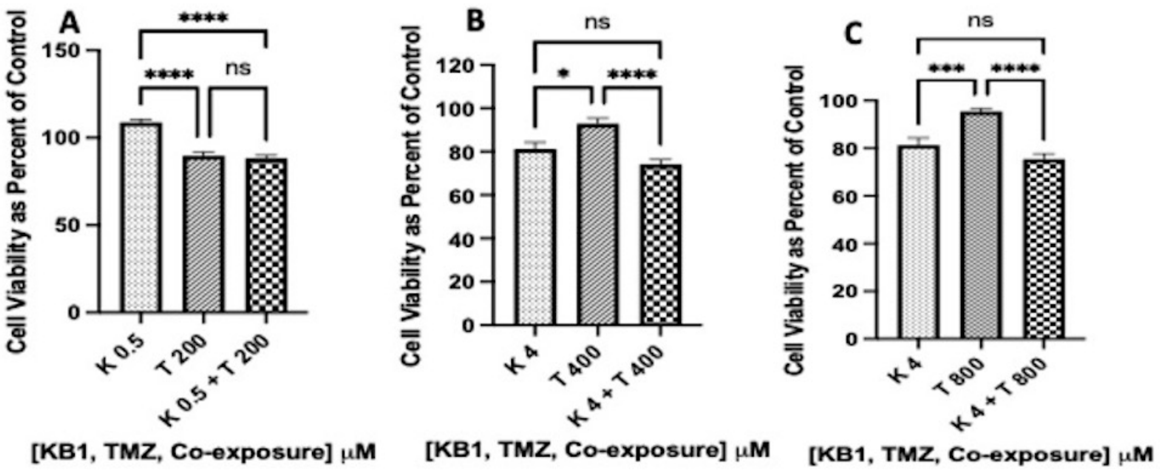

Figure S5. Synthetic Kalata B1 did not significantly increase TMZ-induced cytotoxicity in U-87 MG glioblastoma cell culture when using the following co-exposure concentrations: Kalata B1 (K) 0.5 µM + Temozolomide (T) 200 µM, K 4 µM + T 400 µM, and K 4 µM + T 800 µM.

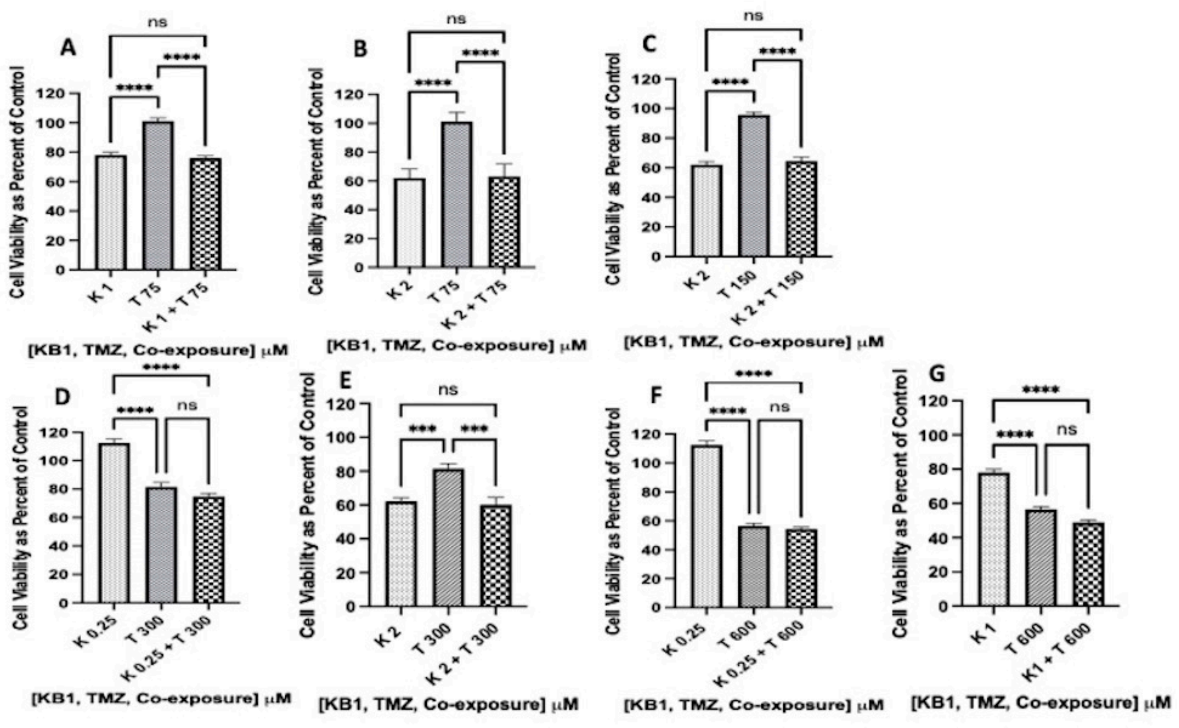

Figure S6. Synthetic Kalata B1 did not significantly increase TMZ-induced cytotoxicity in T-98 glioblastoma cell culture when using the following co-exposure concentrations: Kalata B1 (K) 1 μM + Temozolomide 75 μM, K 2 μM + T 75 μM, K 2 μM + T 150 μM, K 0.25 + T 300 μM, K 2 μM + T 300 μM, K 1 μM + T 600 μM.

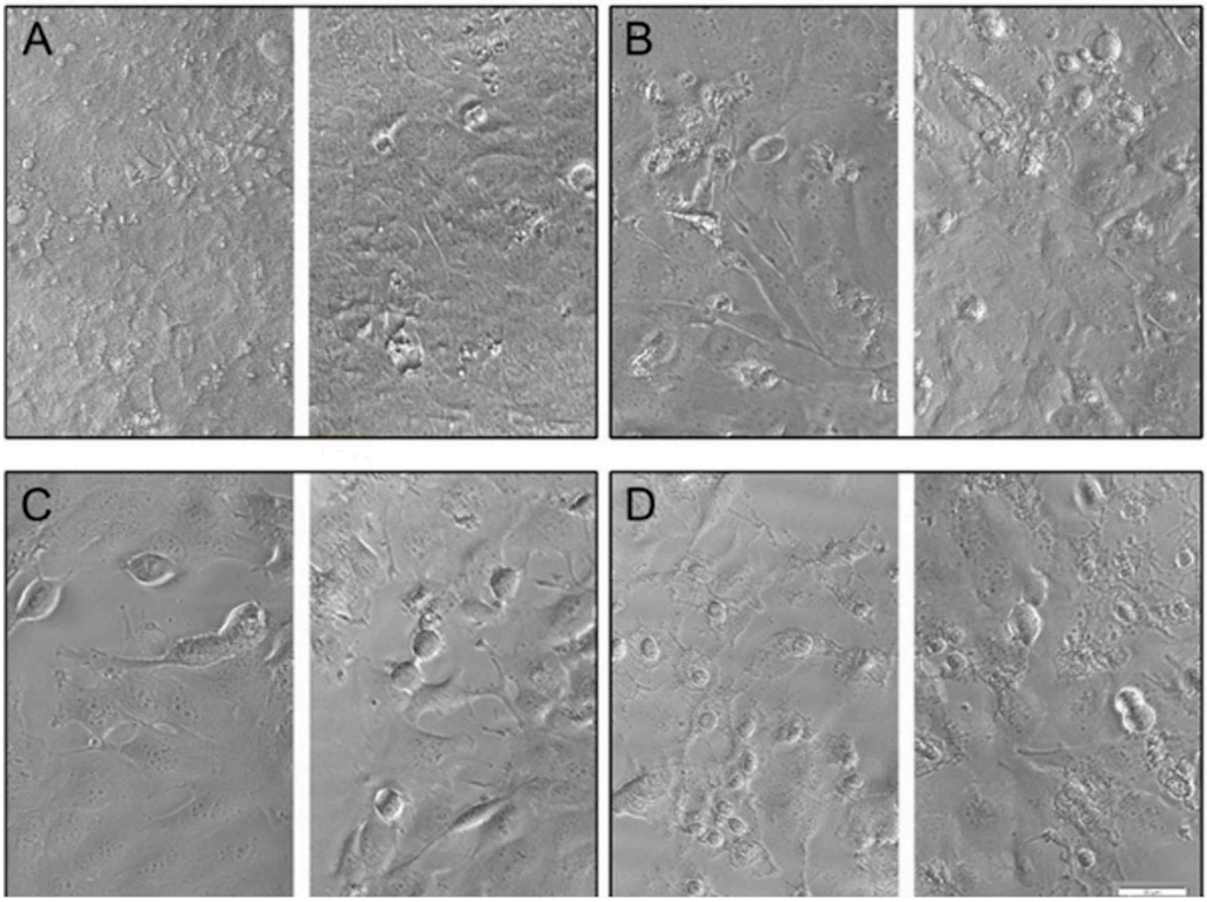

Figure S7. Light microscopy of T-98 cultures treated with Kalata B1 and temozolomide. Panels are as follows: (A) no treatment/control; B) 150 μM temozolomide (TMZ); C) synthetic Kalata B1 0.5 μM; and D) 150 μM TMZ + 0.5 μM synthetic Kalata B1 for 72 hrs. Imaged on an Olympus IX83 inverted microscope equipped with an Olympus XM10 camera and acquired in cellSens Standard. Images were adjusted for exposure in Adobe Photoshop 23.4.2 using "Levels" and cropped for consistency and to enable ease of comparison. T-98 cells were plated at 6000 cells/well in a 96-well plate and allowed to adhere overnight prior to treatment. Images are representative of triplicate incubations. Bar is 50 um. 20 x magnification.
